# Dependence of Mesomorphic Behaviour of Methylene‐Linked Dimers and the Stability of the N_TB_/N_X_ Phase upon Choice of Mesogenic Units and Terminal Chain Length

**DOI:** 10.1002/chem.201601146

**Published:** 2016-05-31

**Authors:** Richard J. Mandle, John W. Goodby

**Affiliations:** ^1^ Department of ChemistryUniversity of YorkYorkYO10 5DDUK

**Keywords:** bimesogens, liquid-crystalline dimers, mesomorphism, nematic phase, X-ray diffraction

## Abstract

Twelve symmetrical dimeric materials consisting of a nonamethylene (C9) spacer and either phenyl 4‐(4′‐alkylphenyl)benzoate, phenyl 4‐(4′‐alkylcyclohexyl)benzoate or phenyl 4‐(4′‐alkylbicyclohexyl)carboxylate mesogenic units were prepared and their mesogenic behaviour characterised by POM, DSC and XRD. All of the materials exhibited nematic phases with clearing points in excess of 200 °C. Four compounds were found to exhibit the twist‐bend nematic phase, with one material exhibiting a transition from the N_TB_ phase into an anticlinic smectic ‘X’ phase. Across all three series of compounds the length of terminal chain is seen to dictate, to some degree, the type of mesophase formed: shorter terminal chains favour nematic and N_TB_ mesophases, whereas longer terminal aliphatic chains were found to promote smectic phases.

## Introduction

In recent years there has been a resurgence of interest in liquid‐crystal dimers and bimesogens, driven by interest in wide temperature range blue phases,^1^, ^2^ flexoelectric behaviour^3^, ^4^, ^5^ and the ability of some dimeric materials to exhibit a lower‐temperature mesophase lacking lamellar organisation thereby being described as nematic.^6^, ^7^, ^8^, ^9^, ^10^, ^11^, ^12^, ^13^, ^14^, ^15^, ^16^, ^17^, ^18^ This lower temperature ‘nematic’ phase is denoted N_X_ or N_TB_,^6^, ^7^, ^8^, ^9^, ^10^, ^11^, ^12^, ^13^, ^14^, ^15^, ^16^, ^17^, ^18^ in which ‘X’ and ‘TB’ refer to unknown and twist‐bend, respectively. The local structure of the N_X_/N_TB_ phase is still hotly debated, with the heliconical ‘twist‐bend’ model proposed independently by Meyer^19^ and Dozov^20^ supported by ^2^H NMR studies, measurement of the electroclinic effect, freeze‐fracture transmission electron microscopy (FFTEM) and carbon K‐edge SAXS.^21^, ^22^, ^23^, ^24^ All three of these methods suggest a local helical structure of extremely tight pitch, in the region of 8 nm for the well‐studied material CB7CB {4′,4′′′‐(heptane‐1,7‐diyl)bis([1,1′‐biphenyl]‐4‐carbonitrile)}.^21^, ^22^, ^23^, ^24^, ^25^ Polarised Raman spectroscopy has been used to measure order parameters in the nematic and N_TB_ phases.^26^

However, this view of the local structure has also been disputed, with a growing body of experimental evidence now interpreted to be counter the heliconical model. Specifically, Hoffmann used a novel ^2^H NMR experiment to show that a helix is not present in the N_TB_/N_X_ phase.^27^ Gorecka et al. demonstrated that similar periodic length scales exist in the solid state of 4′,4′′′‐(nonane‐1,9‐diyl)bis(([1,1′‐biphenyl]‐4‐carbonitrile)) (CB9CB) as those measured by FFTEM, calling the validity of this method into question.^28^ In the original model proposed by Dozov the spontaneous twit‐bend deformation of the nematic director is a consequence of the bend elastic constant K_33_ falling below zero, conversely, experimental studies have shown that this is not the case for CB7CB.^29^ Most recently, the observation of direct isotropic liquid to N_TB_/N_X_ phases has provided a further challenge to future theoretical treatments.^30^, ^31^

There is significant interest not only in the local and bulk structures of the N_TB_ phase, but also the molecular features that give rise to this unique phase of matter. Although predominantly exhibited by methylene‐linked bimesogens, the N_TB_ phase has also been reported in bent‐coresystems,^11^ in covalently and hydrogen bonded trimers^16^, ^17^ and recently in a linear tetramer.^32^ Furthermore, methylene linking groups have been found not to be a prerequisite for N_TB_ phase formation, with both imine,^6^ ether, and mixed ether–ester materials also shown to exhibit this phase.^13^, ^14^, ^33^ Although the N_TB_ phase is known to exhibit local spontaneous chirality,^21^ it has also been observed in neat chiral materials^34^, ^35^ and also upon the introduction of a chiral dopant to an achiral host system.^12^, ^30^

**Figure 1:**
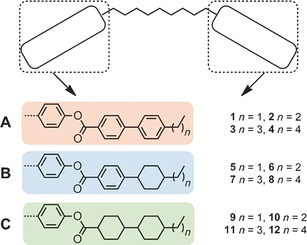
General structure of nonamethylene‐linked dimers and target structures. Experimental details are given in the Supporting Information to this article.

Recently we demonstrated how the length of the terminal alkoxy chain in nonamethylene (C9)‐linked phenyl 4‐alkoxybenzoates dictates the mesophase behaviour of the system, with shorter homologues (<C7) exhibiting nematic and N_TB_ phases and longer homologues (>C7) exhibiting anticlinic smectic C phases.^18^ In this article we present a study of the dependence of mesomorphic behaviour upon the terminal chain length in dimeric materials consisting of a nonamethylene (C9) spacer and either phenyl 4‐(4′‐alkylphenyl)benzoate, phenyl 4‐(4′‐alkylcyclohexyl)benzoate or phenyl 4‐(4′‐alkylbicyclohexyl)carboxylate mesogenic units, and on the degree of incorporation of alicyclic rings into the rigid mesogenic units (Figure 1).

## Experimental Section

Bis{1,9‐(4‐hydroxyphenyl)nonane} and compounds **4**, **8** and **12** were prepared as described previously.^15^, ^18^ Reagents and solvents were obtained from commercial suppliers. Computational chemistry was performed in Gaussian G09 Revision D.01^36^ with output files rendered using QuteMol.^37^ Full experimental details, including synthetic procedures and chemical characterisation, are provided in the accompanying Supporting Information.

## Results and Discussion

The mesomorphic properties of compounds **1**–**12** were studied by a combination of polarised optical microscopy (POM) and differential scanning calorimetry (DSC). Phase identifications were made based on textures observed by POM, assisted where appropriate with small angle X‐ray scattering data (SAXS).The transition temperatures (°C) and enthalpies of transition (kJ mol^−1^) are given for each series of compounds in Tables 1 to 3, respectively.

**Figure 2:**
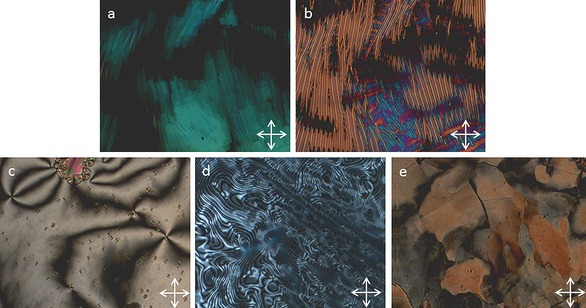
Photomicrographs (×100) of the blocky texture of the N_TB_ phase of **2** (a, 162 °C), the rope texture of the N_TB_ phase of **2** (b, 142 °C), a small focal‐conic defect and the schlieren texture of the smectic C_A_ phase of **4** (c, 190 °C), the schlieren texture of the ‘Sm_X_’ phase of **2** (d, 135 °C) and the mosaic texture of the B phase of **4** (e, 100 °C)

Table 1 presents the mesomorphic behaviour of four nonamethylene‐linked phenyl 4‐(4‐alkylphenyl)benzoate dimers. All four materials exhibited enantiotropic nematic phases. Compounds **1** and **2** both exhibit enantiotropic N_TB_ phases, the latter material also exhibiting an additional smectic phase.^38^ Upon increasing the length of the terminal chain from propyl to butyl, the N_TB_ phase is suppressed. The lower temperature ‘Sm_X_’ phase exhibited by **2** is not seen, with a direct nematic to anticlinic smectic C transition being observed instead. A further increase in the terminal chain length from butyl to pentyl, shows that compound **4**, affords a further increase in the onset temperature of the smectic C_A_ phase with an additional monotropic B phase observed on cooling. Photomicrographs of the defect textures of the mesophases are presented in Figure 2.

**Table 1:** Transition temperatures [°C] and associated enthalpies of transition [kJ mol^−1^] for compounds **1**–**4**.^17^, ^38^

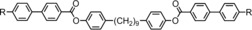														
Compd.	R	Cr		B		SmC_A_		Sm_X_		N_TB_		N		Iso
**1**	C_2_H_5_	•	153.0 [31.49]	–	–	–	–	–	–	•	155.5 [0.01]	•	241.4 [1.30]	•
**2**	C_3_H_7_	•	129.3 [30.19]	–	–	–	–	•	137.1 [1.63]	•	167.2 [0.01]	•	244.9 [1.36]	•
**3**	C_4_H_9_	•	117.3 [28.06]	–	–	•	172.8 [3.87]	–	–	–	–	•	228.1 [1.23]	•
**4**	C_5_H_11_	•	128.0 [25.64]	(•	106.1) [4.94]	•	191.7 [6.07]	–	–	–	–	•	226.1 [2.32]	•

**Figure 3:**
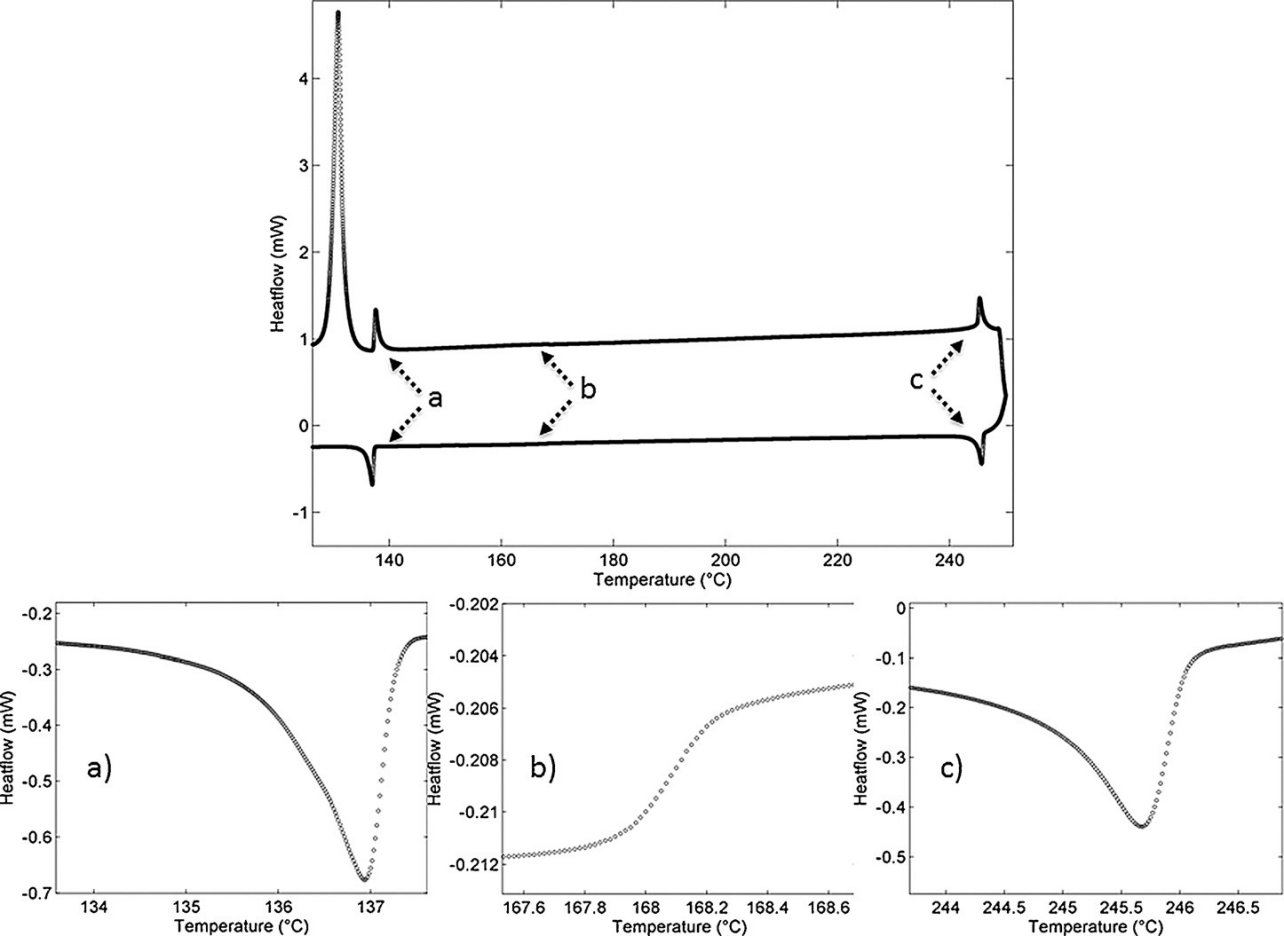
DSC trace of compound **2** on first heating (top, positive heatflow) and cooling (bottom, negative heatflow) with an expansion showing the Sm_X_–N_TB_ transition (a), the N_TB_–N transition (b) and the N–Iso transition (c).

Cooling compounds **1** and **2** from the nematic to N_TB_ phase yielded the typically broken stepped texture that is characteristic for this phase (Figure 2 a). Further cooling of the N_TB_ phase yields a parabolic texture that coalesces to the rope texture as described previously (Figure 2 b).^12^ The smectic C phase of compounds **3** and **4** was identified as having anticlinic layer ordering from the observation of both 2‐ and 4‐brush dispirations in the schlieren texture (Figure 2 c). The B phase of compound **4** was identified by its mosaic texture (Figure 2 e), which shows the presence of long range ordering. The DSC thermogram of the first heat and cooling cycle of compound **2** is shown in Figure 3. Previously it has been reported that, for certain materials, notably CB11CB, the nematic to N_TB_ phase transition is rather broad and appears second order in contrast to the sharp peak associated with the nematic to isotropic transition.^12^ Both compounds **1** and **2** exhibit this pseudo second order nematic to N_TB_ phase transition that is somewhat reminiscent of a glass transition and is indicative of kinetic processes occurring at the phase transition, which may be indicative of the N_TB_ phase having a helical superstructure. Such behaviour is analogous to that of the cubic ‘smectic D’ phase, which possesses a bicontinuous cubic structure and also exhibits a columnar phase depending on the rate of change of temperature at the phase transition.^39^

**Figure 4:**
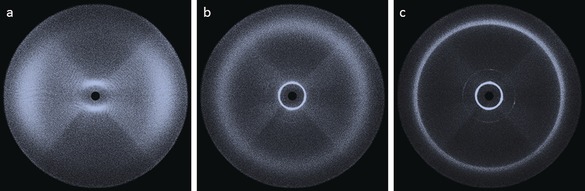
Two‐dimensional X‐ray scattering patterns for compound **4** in the nematic phase (a, 209 °C), the smectic C_A_ phase (b, 165 °C) and the B phase (c, 94 °C).

We elected to use small‐angle X‐ray scattering (SAXS) to study the N–SmC_A_–B polymorphism exhibited by compound **4**. The nematic phase was well‐aligned by the magnetic field and gave a typical diffuse small‐angle scattering pattern (Figure 4 a). Conversely, the SmC_A_ and B phases were unaligned by the field and so the resulting SAXS patterns are not diagnostic of the local structure for either mesophase. Qualitatively the definition of the small‐angle peak can be seen to increase sharply moving across the N–SmC_A_–B phase sequence. Additional second‐order scattering in the small‐angle region can also be seen in Figure 4 c, which indicates the long‐range in‐plane correlation length of the B phase. The scattered intensity in the wide‐angle region (small values of d) relates to the average lateral separation between molecules, thus, it is to be expected that this presents as diffuse in the nematic phase. This lack of definition at wide angles is typical of a nematic phase, and only a small increase in definition is observed on entering the smectic C_A_ phase. There is a local hexagonal packing of molecules in the B phase and this leads to a notable increase in the definition of the wide‐angle region (Figure 4 c).

The layer spacing of the smectic C_A_ mesophase exhibited by compounds **3** and **4** was measured by small‐angle X‐ray scattering. In both materials the layer spacing was found to be virtually temperature independent, with measured values of 23.3 Å for **3** and 24.2 for **4**. These values correspond to *d*/*l* ratios (i.e., the ratio of layer‐spacing to molecular lengths, calculated on geometry optimised at the B3LYP/6–31G(d) level of DFT) of 0.55 for **3** and 0.53 for **4**, indicating that the smectic C_A_ phase in both materials is extensively intercalated. For compound **4**, a temperature‐independent layer spacing of 24.3 Å was observed in the B phase which leads to a *d*/*l* ratio of 0.54.

**Figure 5:**
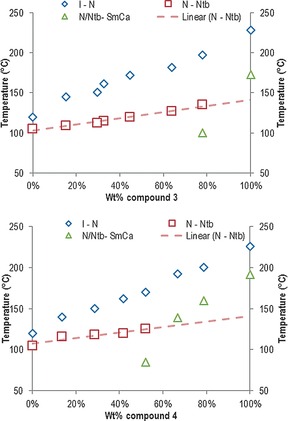
Binary phase diagrams for compound **3** (top) and compound **4** (bottom) with CB9CB.

As compounds **3** and **4** exhibit a direct nematic to smectic C_A_ phase transition without an intervening twist‐bend nematic phase we elected to construct phase diagrams (Figure 5) between these two materials and the well‐studied bimesogen CB9CB,^9^ which exhibits an enantiotropic N_TB_ phase so as to allow determination of the virtual nematic to N_TB_ transition temperatures for these compounds.

Binary mixtures between **3** and CB9CB exhibit the twist‐bend nematic phase up to relatively high concentrations (80 wt % of **3**). In the phase diagram between **4** and CB9CB, the N_TB_ can be observed up to around 50 wt % of **4**, after which a direct nematic to smectic C_A_ transition is observed. Linear fitting of the N_TB_–N transition temperature as a function of concentration allows extrapolation of the virtual nematic to twist‐bend nematic transition temperatures for **3** and **4**. This was determined to be 141.7 and 141.1 °C for compounds **3** and **4**, respectively. Notably, this virtual transition temperature occurs at a lower temperature than that of the N–SmC_A_ transition and hence is not observed in the pure materials. However, the virtual N_TB_–N transition temperatures for **3** and **4** are still significantly higher than that measured for CB9CB (104.8 °C).

**Figure 6:**
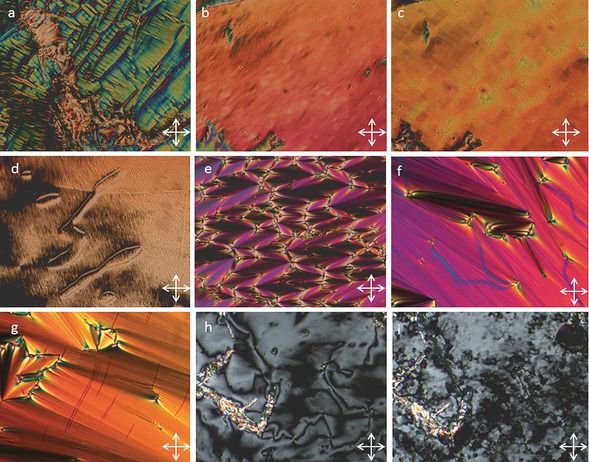
Photomicrographs (×100) of the defect texture of the N_TB_ phase of compound **5** (a, 110 °C), the texture of a planar aligned region of the nematic phase of **5** (b, 126 °C), and the blocky texture of the N_TB_ phase in **5** observed in approximately the same region as that for the sample shown in b (c, 121 °C), the schlieren texture of the nematic phase of **6** (d, 162 °C) and focal‐conic defects for the smectic C_A_ phase of **7** (e, 140 °C), focal‐conic defects in the smectic C_A_ phase of **8** (f, 160 °C), the paramorphotic focal‐conic texture of the B phase of **8** (g, 113 °C), the schlieren texture of the SmC_A_ phase of **8** (h, 122 °C) and the coarse mosaic texture of the B phase of **8** (i, 111 °C). Photomicrographs ‘h’ and ‘i’ are approximately for the same area of the sample.

Table 2 presents the mesomorphic behaviour of four nonamethylene‐linked phenyl *trans* 4‐(4‐alkylcycohexyl)benzoate dimers. As with compounds **1**–**4**, all materials exhibited enantiotropic nematic phases. The two homologues bearing shorter terminal chains (**5** and **6**, ethyl and propyl, respectively) exhibited an additional twist‐bend nematic phase. Compounds **7** and **8** do not exhibit the N_TB_ phase, instead a direct nematic to anticlinic smectic C phase was observed. Both the nematic and N_TB_ phases can be identified from their optical textures, with photomicrographs presented in Figure 6.

**Table 2:** Transition temperatures [°C] and associated enthalpies of transition [kJ mol^−1^] for compounds **5**–**8**.^17^

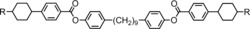												
Compd.	R	Cr		B		SmC_A_		N_TB_		N		Iso
**5**	C_2_H_5_	•	126.1 [29.42]	–	–	–	–	(•	124.5) [0.01]	•	214.4 [1.40]	•
**6**	C_3_H_7_	•	125.5 [24.41]	–	–	–	–	•	141.6 [0.01]	•	233.6 [1.54]	•
**7**	C_4_H_9_	•	108.6 [50.78]	(•	96.2) [3.29]	•	149.3 [3.17]	–	–	•	228.3 [2.01]	•
**8**	C_5_H_11_	•	94.8 [42.33]	•	114.6 [3.33]	•	171.7 [2.99]	–	–	•	226.3 [1.99]	•

**Figure 7:**
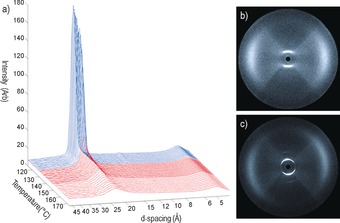
A plot of integrated scattering intensities for a magnetically aligned sample of compound **7** as a function of temperature (a). The highest temperature is 173 °C and the lowest 114 °C, with data recorded in 1 °C steps (2 °C steps in the region 173–145 °C). Small‐angle scattering patterns for compound **7** in the nematic phase (b, 151 °C) and in the smectic C_A_ phase (c, 125 °C).

The smectic C phase was confirmed as possessing anticlinic layer ordering by observation of 2‐ and 4‐ brush disparations in the schlieren texture (shown in Figure 6 h compound **8**). For compounds **7** and **8** an additional phase transition was observed by DSC at 96.2 and 114.6 °C, respectively, which was classified as a B phase. Upon cooling from the anticlinic smectic C phase a transitory paramorphotic focal‐conic texture was observed (Figure 7 g) as the schlieren texture of the SmC_A_ phase gave way to a coarse mosaic texture (Figure 6 h and i).

We next elected to study compound **7** by SAXS (Figure 7). The direct N–SmC_A_ transition for compound **7** means that it was possible to partially align the smectic C_A_ phase in a magnetic field, although alignment was insufficient for quantitative determination of orientational order parameters. Additionally, the well‐known relationship between the orientational distribution function and scattered intensity in the wide‐angle region assumes that the molecules have cylindrical symmetry.^40^, ^41^ As ‘bent’ bimesogens are molecularly biaxial it is unclear if the results obtained by this method are physically meaningful. As with X‐ray studies on compound **4** the diffuse wide‐angle scattering in the nematic phase, which corresponds to the average lateral distance between adjacent molecules, grows somewhat more defined upon entering the smectic C_A_ phase. This is accompanied by a decrease in the d‐spacing of the peak corresponding to the wide‐angle scattering, which is a consequence of the average lateral molecular separation being larger in the nematic that in the smectic C_A_ phase. The layer spacing, determined from the d‐spacing of the sharp peak in the small angle region of the SmC_A_ phase was found to have a temperature‐ independent value of 23.60 Å, which corresponds to 0.53 molecular lengths (calculated to be 44.92 Å at the B3LYP/6–31G(d) level of DFT).

For compound **8** the SmC_A_ layer spacing, as determined by SAXS, was found to have a temperature‐independent value of 24.2 Å, corresponding to a *d*/*l* ratio of 0.55. Compounds **3**, **4**, **7**, and **8** all exhibit SmC_A_ phases with temperature‐independent *d*/*l* ratios in the region of 0.50–0.55, indicating that in all cases the smectic C_A_ phase is extensively intercalated irrespective of terminal chain length or the type of mesogenic unit employed. The B phase exhibited by compounds **7** and **8** was also studied by SAXS, although the partial alignment present in the smectic C_A_ phase of both materials was lost upon cooling into the B phase. The temperature independent layer spacing of the smectic B phase was measured to be 23.2 and 23.5 for **7** and **8**, respectively, corresponding to *d*/*l* ratios of 0.52 for **7** and 0.54 for **8**.

**Figure 8:**
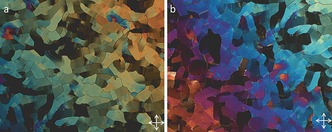
Photomicrographs (×100) of the mosaic texture of the B phase of **9** at 165 (a) and 160 °C (b).

The mesomorphic properties of four nonamethylene‐linked phenyl *trans*,*trans*4‐(4‐alkylcycohexyl)cyclohexylcarboxylate dimers are presented in Table 3. Unlike the two previous families of compounds, the shorter homologues do not exhibit the N_TB_ phase, but instead show direct nematic to B phase transitions. Photomicrographs of the defect texture of the B phase exhibited by compound **9** are shown in Figure 8. The identity of the B phase of **12** has been confirmed previously by miscibility studies with 65OBC.^18^

**Table 3:** Transition temperatures [°C] and associated enthalpies of transition [kJ mol^−1^] for compounds **9**–**12**.^17^

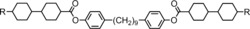												
Compd.	R	Cr		B		SmC_A_		SmA		N		Iso
**9**	C_2_H_5_	•	69.0 [31.67]	•	189.2 [13.05]	–	–	–	–	•	254.2 [2.93]	•
**10**	C_3_H_7_	•	77.0 [30.36]	•	174.3 [11.16]	–	–	–	–	•	232.3 [1.56]	•
**11**	C_4_H_9_	•	82.9 [23.91]	•	199.5 [4.03]	–	–	•	200.3 [4.02]	•	245.3 [0.93]	•
**12**	C_5_H_11_	•	85.5 [34.34]	•	199.1 [3.82]	•	202.8 [0.66]	•	208.1 [1.16]	•	243.3 [1.53]	•

**Figure 9:**
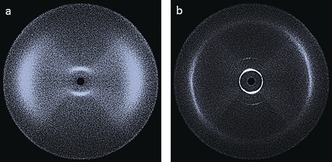
Two‐dimensional X‐ray scattering patterns for compound **10** in the nematic phase (a, 184 °C), and the B phase (b, 163 °C).

The direct nematic to B phase transition in compounds **9** and **10** results in the B phase exhibiting its natural textures, that is, homeotropic and mosaic, rather than the paramorphotic focal‐conic textures observed for the other compounds in this work. Compound **10** was studied by small‐angle X‐ray scattering (Figure 9). Despite the smectic phase being only partially aligned, the sharp increase in the definition of the wide‐angle region is commensurate with the onset of hexagonal close packing in the B phase, whereas the onset of a second‐order reflection in the small angle region is indicative of a long‐range out of plane correlation length.

**Figure 10:**
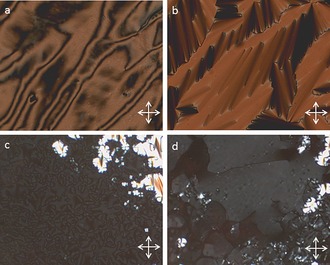
Photomicrographs (×100) of the nematic schlieren texture of **12** (a, 222 °C), the smectic A phase of **12** (b, 203 °C), the schlieren and focal‐conic textures of the smectic C_A_ phase of **12** (c, 199.9 °C), and the mosaic and paramorphotic focal‐conic texture of the B phase of **12** (d, 190 °C).

Compound **11**, which possesses butyl terminal chains, exhibits an additional smectic A phase over a short temperature range (<1 °C), whereas compound **12** exhibits an additional smectic C_A_ phase. Photomicrographs for the textures of the mesophases exhibited by **12** are given in Figure 10.The smectic mesophases of compounds **11** and **12** were studied by SAXS. The layer spacing for the smectic B phase exhibited by compounds **11** and **12** was measured to be 23.64 and 24.68 Å, respectively, with both values found to be temperature independent. Using molecular lengths calculated at the B3LYP/6–31G(d) level of DFT (43.8 Å for **11** and 44.5 Å for **12**) the measured layer spacings for **11** and **12** correspond to *d*/*l* ratios of 0.54 and 0.55, respectively, indicating that as with compounds **4** and **8** the B phase exhibited by methylene linked bimesogens is heavily intercalated.

## Discussion

The present results show that in bimesogens the specific mesophases exhibited can be controlled by employing differing mesogenic units. All twelve dimeric materials exhibited a nematic mesophase, with those incorporating phenyl biphenylbenzoate (**1**–**4**) or phenyl cyclohexylbenzoate (**5**–**8**) mesogenic units exhibiting additional twist‐bend nematic or tilted smectic mesophases. The behaviour of compound **1**–**8** is in contrast to bimesogens incorporating phenyl bicyclohexylcarboxylate mesogenic units; compounds **9** and **10** exhibit nematic and B phases, with longer homologues also exhibiting additional SmA (**11**) or SmA and SmC_A_ (**12**) phases. The ability to control the mesophases exhibited by a given bimesogen by chemically altering the mesogenic units is seemingly analogous to that of typical calamitic materials, where biphenyl benzoates will tend to give nematic and tilted phases and the analogous bicyclohexyl materials will preferentially exhibit higher‐order smectic phases.^39^ Experimental observations of phase transitions between the N_TB_/N_X_ phase and smectic mesophases are still rare, and as yet no theoretical treatments of such events have been provided.

**Figure 11:**
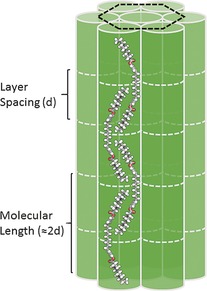
Model of hexagonal close packing of bimesogens, in this case compound **9** in its B3LYP/6‐31G(d) minimised geometry, in the B phase exhibited by compounds **4** and **7**–**12**.

Figure 11 shows a proposed model for packing of methylene linked bimesogens with odd spacer parity in the B phase. In keeping with experimentally determined layer spacings and *d*/*l* ratios in the B phase, we propose that the molecules are strongly intercalated with a smectic layer spacing of approximately 0.5 molecular lengths. Due to the hexagonal close packing of the molecules it is expected that rotation about the long axis can only be achieved through cooperative motion, as for low molar mass materials.^39^ As far as we are aware, these are the first examples of odd‐parity bimesogens exhibiting B phases.

The *d*/*l* ratios for the smectic C_A_ phases of **3**, **4**, **7**, and **8** are all in the region of 0.50–0.55, indicating that the mesophase is highly intercalated. Similarly for the B phases observed in compounds **4**, **8**, **10**, **11** and **12** the *d*/*l* ratios were in the region of 0.50–0.55. While the type of mesophase exhibited by bimesogens can be dictated to some extent by informed choice of mesogenic unit and length of terminal group with each giving different layer spacings due to the differing molecular length, ultimately the *d*/*l* ratio is unchanged.

As shown in Table 4the associated entropies and enthalpies of transition (both determined by differential scanning calorimetry with a heat/cool rate of 10 °C min^−1^) for nematic to N_TB_ transition in compounds **1**, **2**, **5** and **6** are approximately two orders of magnitude lower than those for the nematic to isotropic transition in the same materials. There is a slight increase in the scaled transition temperatures (*T*_NTB–N_/*T*_N–Iso_) with increasing terminal chain length; however, further increases yield direct nematic to smectic phase transitions as discussed previously. The values of the associated enthalpies and entropies of the N–I and N_TB_–N transitions increase when the mesogenic unit is changed from phenyl biphenyl carboxylate (compounds **1** and **2**) to a phenyl cyclohexylbenzoate (**5** and **6**), indicating that the choice of mesogenic unit not only impacts upon the specific thermal behaviour of a given material but also on its transitional properties. Comparison of the associated enthalpies and entropies of transition of **1**, **2**, **5** and **6** with those of CB11CB^12^ reveals that the isotropisation occurs with comparable values of associated enthalpy and entropy; however, at the N_TB_–N transition the associated enthalpy and entropy of transition are significantly larger for CB11CB than for the compounds presented in this work. As the associated enthalpy (and thus entropy) of a transition are often taken to be indicative of the change in local structure at a phase transition, this discrepancy suggests that there is a bigger shift in mesophase structure at the N_TB_–N transition of CB11CB than for **1**, **2**, **5** and **6**. How this is borne out in terms of the properties of the N_TB_ phase is presently unclear.

**Table 4:** Associated enthalpies of transition [kJ mol^−1^] with corresponding standard deviations (SD), dimensionless entropies of transition and scaled N/N_TB_ transition temperatures for materials exhibiting a twist‐bend nematic phase along with data for CB11CB for comparative purposes.^12^ Enthalpy and entropy values are the mean average obtained from six heat/cool cycles, whereas other values in this manuscript are the average of just two cycles.

No.	Δ*H* [kJ mol^−1^]				*ΔS/R*		*T*_NTB–N_/*T*_N–Iso_
	N_TB_–N	SD	N–Iso	SD	N_TB_–N	N–Iso	
**1**	1.13e‐2	0.0019	1.299	0.0096	3.16e‐3	0.304	0.644
**2**	1.05e‐2	0.0105	1.379	0.0054	2.88e‐3	0.320	0.683
**5**	1.44e‐2	0.0008	1.364	0.0043	4.34e‐3	0.337	0.581
**6**	1.30e‐2	0.0011	1.551	0.0081	3.76e‐3	0.38	0.606
CB11CB	3.51e‐2	–	1.541	–	1.106e‐2	0.465	0.865

## Conclusion

We have demonstrated that the N_TB_ phase becomes less stable in comparison to intercalated smectic phases as the proportionality of σ‐ to π‐bonded structuring is increased. This is consistent with conventional calamitic low molar mass materials where smectic mesophases become stabilised with increasing chain length. However, it is also known that the presence of alicyclic rings systems also promotes nematic behaviour particularly when substituted with short aliphatic chains, but as the terminal substituent is extended their behaviour becomes more in keeping with long aliphatic chains. This will lead to stabilisation of smectic phase behaviour and that of the B phase in particular and thus is similar to the hydrophobic effect found for classical amphiphilic behaviour.^42^

## Supporting information

As a service to our authors and readers, this journal provides supporting information supplied by the authors. Such materials are peer reviewed and may be re‐organized for online delivery, but are not copy‐edited or typeset. Technical support issues arising from supporting information (other than missing files) should be addressed to the authors.

Supplementary
