# Supplementary material for: Dependence of Mesomorphic Behaviour of Methylene‐Linked Dimers and the Stability of the NTB/NX Phase upon Choice of Mesogenic Units and Terminal Chain Length
Source: Chemistry. 2016 May 31;22(27):9366–74. doi: 10.1002/chem.201601146 (PMC5132152; doi:10.1002/chem.201601146)
Supplement: Supplementary file 1 — Supplementary [file CHEM-22-9366-s001.pdf]

CHEMISTRY
A European Journal

Supporting Information

Dependence of Mesomorphic Behaviour of Methylene-Linked Dimers and the Stability of the N_{TB}/N_X Phase upon Choice of Mesogenic Units and Terminal Chain Length

Richard J. Mandle* and John W. Goodby[a]

chem_201601146_sm_miscellaneous_information.pdf

1.1 General Methods.

Bis 1,9-(4-Hydroxyphenyl)nonane, and compounds 4, 8, 12 were prepared as described previously. [1, 2] 4'-Alkylbiphenyl-4-carboxylic acids, *trans* 4'-alkylcyclohexylbenzoic acids and *trans trans* 4'-alkylcyclohexylcyclohexane carboxlic acids were purchased from Synthon Gmbh or available in-house. Compounds **4**, **8** and **12** were prepared as described previously. [2] 1-Ethyl-3-(3-dimethylaminopropyl)carbodiimide (EDAC) was purchased from Carbosynth UK, *N,N*-dimethylaminopyridine (DMAP) was purchased from Sigma Aldrich. Solvents were purchased from Fisher Scientific UK and were dried *via* passage over activated alumina prior to use. Reactions were monitored by thin layer chromatography (TLC) using an appropriate solvent system. Silica coated aluminium TLC plates used were purchased from Merck (Kieselgel 60 F-254) and visualised using either UV light (254 nm and 365 nm), or by oxidation with either iodine or aqueous potassium permanganate solution. Yields refer to chromatographically (HPLC) and spectroscopically (^{1}H NMR, ^{13}C NMR) homogenous material.

NMR spectra were recorded on a JEOL ECX spectrometer operating at 400 MHz (^{1}H), 100.5 MHz (^{13}C) and referenced to either TMS or residual protic solvent (^{1}H NMR). Mass spectra were recorded on a Bruker micrOTOF MS-Agilent series 1200LC spectrometer. FT-IR spectroscopy was performed using a Shimadzu IR Prestige-21 with Specac Golden Gate diamond ATR IR insert. Polarised optical microscopy was performed on a Zeiss Axioskop 40Pol microscope using a Mettler FP82HT hotstage controlled by a Mettler FP90 central processor. Photomicrographs were captured *via* either an InfinityX-21 digital camera or a Sony NEX 5R mirrorless digital camera mounted atop the microscope. Differential scanning calorimetry was performed on a Mettler DSC822^{e} fitted with an autosampler operating with Mettler Star^{e} software and calibrated before use against an indium standard (onset = 156.55 ± 0.2 °C, ΔH = 28.45 ± 0.40 Jg^{-1}) under an atmosphere of dry nitrogen. Computational chemistry was performed using Gaussian G09 revision d01 on the York Advanced Research Computing Cluster (YARCC) as described in the text. [3]

Small angle X-ray diffraction was performed using a Bruker D8 Discover equipped with a temperature controlled, bored graphite rod furnace, custom built at the University of York. The radiation used was copper Kα (λ = 0.154056 nm) from a 1 μS microfocus source. Diffraction patterns were recorded on a 2048x2048 pixel Bruker VANTEC 500 area detector. Samples were filled into 1mm capillary tubes and aligned with a pair of 1T magnets. Diffraction patterns were collected as a function of temperature and the data processed using Matlab. Raw data are available upon request from the University of York data catalogue at DOI: 10.15124/81c5cad4-a5f4-496b-b51d-05f0031ecd9a

1.2. General Synthetic Procedure

A suspension of *bis* 1,9-(4-Hydroxyphenyl)nonane (312 mg, 1 mmol), carboxylic acid (3 mmol), EDAC (3 mmol) and DMAP (25 mg) in dry dichloromethane (5 ml) was stirred until complete consumption of *bis* 1,9-(4-

Hydroxyphenyl)nonane as evidenced by TLC (typically within 2 h). The crude materials were purified by dry vacuum flash chromatography [4, 5] with varying ratios of DCM/hexanes as the eluent, followed by recrystalisation from ethanol/THF (varying ratios) to yield the target compounds in yields of 65 – 80% of theory.

1.3. Material Characterisation

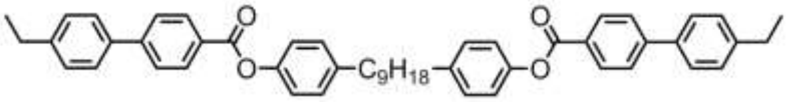

Nonane-1,9-diyl*bis*(4,1-phenylene) bis(4'-ethyl-[1,1'-biphenyl]-4-carboxylate), 1

Yield:	567 mg (78 %)
^{1}H NMR (400 MHz, $CDCl_3$):	1.29 (6H, t, *J* = 7.8 Hz, $\underline{CH_3}$-CH_2-Ar), 1.29 – 1.38 (10H, m, Ar-CH_2-CH_2-$\underline{(CH_2)_5}$-CH_2-CH_2-Ar), 1.63 (4H, quintet, *J* = 6.9 Hz, Ar-CH_2-$\underline{CH_2}$-$(CH_2)_5$-$\underline{CH_2}$-CH_2-Ar), 2.63 (4H, t, *J* = 6.8 Hz, Ar-$\underline{CH_2}$-$(CH_2)_7$-$\underline{CH_2}$-Ar), 2.72 (4H, quartet, *J* = 7.8 Hz, CH_3-$\underline{CH_2}$-Ar), 7.12 (4H, d, *J* = 8.7 Hz, Ar*H*), 7.23 (4H, d, *J* = 8.7 Hz, Ar*H*), 7.31 (4H, d, *J* = 8.7 Hz, Ar*H*), 7.58 (4H, d, *J* = 8.7 Hz, Ar*H*), 7.70 (4H, d, *J* = 8.7 Hz, Ar*H*), 8.24 (4H, d, *J* = 8.7 Hz, Ar*H*)
^{13}C NMR (100.5 MHz, $CDCl_3$):	165.27, 148.83, 146.14, 144.60, 140.48, 137.19, 130.63, 129.32, 128.49, 128.05, 127.22, 126.93, 121.32, 35.36, 31.45, 29.46, 29.44, 29.23, 28.55, 15.53.
FT-IR (vmax, cm^{-1}):	692, 729, 761, 825, 873, 1004, 1016, 1076, 1165, 1190, 1263 (C-O stretching), 1398, 1465 (aliphatic C-H bending), 1506 (aromatic C-C stretching), 1602, 1728 (C=O stretching), 2848 (aliphatic C-H stretching), 2926 (aliphatic C-H stretching).
MS (ESI+, m/z):	751.3761 (100%, $C_{51}H_{52}NaO_4$, calcd. for $C_{51}H_{52}NaO_4$ 751.3758, M + Na)

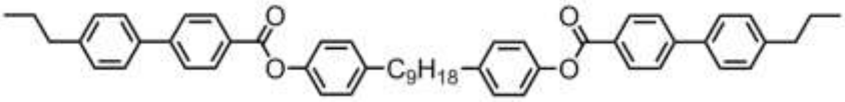

Nonane-1,9-diyl*bis*(4,1-phenylene) bis(4'-propyl-[1,1'-biphenyl]-4-carboxylate), 2

Yield: 520 mg (69 %)

^{1}H NMR (400 MHz, $CDCl_3$): 0.91 (6H, t, J = 7.0 Hz, $C\underline{H}_3$-CH_2-CH_2-Ar), 1.20 – 1.32 (10H, m, Ar-CH_2-CH_2-$\underline{(CH_2)_5}$-CH_2-Ar, 1.50 – 1.68 (8H, m, Ar-CH_2-$\underline{CH_2}$-CH_3 + Ar-CH_2-$\underline{CH_2}$-$(CH_2)_5$-$\underline{CH_2}$-CH_2-Ar), 2.52 – 2.61 (8 H, m [2x t] Ar-$\underline{CH_2}$-CH_2-CH_3 + Ar-$\underline{CH_2}$-CH_2), 7.05 (4H, ddd, *J* = 1.8 Hz, *J* = 2.8 Hz, *J* = 8.5 Hz, Ar*H*), 7.16 (4H, ddd, *J* = 1.8 Hz, *J* = 2.8 Hz, *J* = 8.5 Hz, Ar*H*), 7.22 (4H, ddd, *J* = 1.8 Hz, *J* = 2.1 Hz, *J* = 8.2 Hz, Ar*H*), 7.50 (4H, ddd, *J* = 1.8 Hz, *J* = 2.1 Hz, *J* = 8.2 Hz, Ar*H*), 7.63 (4H, ddd, *J* = 1.8 Hz, *J* = 1.8 Hz, *J* = 8.5 Hz, Ar*H*), 8.17 (4H, ddd, *J* = 1.8 Hz, *J* = 1.8 Hz, *J* = 8.5 Hz, Ar*H*)

^{13}C NMR (100.5 MHz, $CDCl_3$): 165.44, 149.00, 146.31, 143.22, 140.64, 137.33, 130.79, 129.48, 129.25, 128.20, 127.28, 127.09, 121.48, 37.85, 35.53, 31.62, 29.60, 29.46, 29.43, 24.66, 14.01.

FT-IR (vmax, cm^{-1}): 692, 727, 761, 823, 875, 1004, 1016, 1076, 1165, 1190, 1265 (C-O stretching), 1398, 1465 (aliphatic C-H bending), 1506 (aromatic C-C stretching), 1604, 1726 (C=O stretching), 2848 (aliphatic C-H stretching), 2916 (aliphatic C-H stretching).

MS (ESI+, m/z): 779.4033 (100%, $C_{53}H_{56}NaO_4$, calcd. for $C_{53}H_{56}NaO_4$ 779.4071, M + Na)

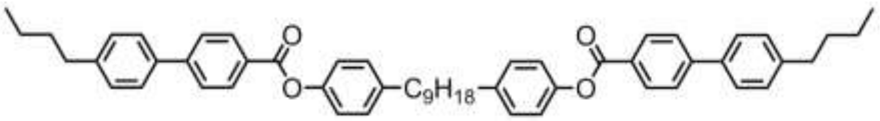

Nonane-1,9-diyl*bis*(4,1-phenylene) *bis*(4'-butyl-[1,1'-biphenyl]-4-carboxylate), 3

Yield:	611 mg (78 %)
^{1}H NMR (400 MHz, $CDCl_3$):	0.95 (6H, t, J = 7.3 Hz, C<u>H</u>$_3$-CH_2-CH_2-CH_2-Ar), 1.27 – 1.44 (14H, m, Ar-CH_2-CH_2-<u>$(CH_2)_5$</u>-CH_2-Ar + CH_3-<u>CH_2</u>-CH_2-CH_2-Ar), 1.58 – 1.69 (8H, m, Ar-CH_2-<u>CH_2</u>-CH_3 + Ar-CH_2-<u>CH_2</u>-$(CH_2)_5$-<u>CH_2</u>-CH_2-Ar), 2.59 – 2.69 (8 H, m [2x t] Ar-<u>CH_2</u>-CH_2-CH_3 + Ar-<u>CH_2</u>-CH_2), 7.12 (4H, ddd, *J* = 2.1 Hz, *J* = 2.8 Hz, *J* = 8.5 Hz, Ar*H*), 7.23 (4H, m, Ar*H*), 7.28 (4H, m, Ar*H*), 7.56 (4H, m, Ar*H*), 7.70 (4H, m, Ar*H*), 8.23 (4H, m, Ar*H*),
^{13}C NMR (100.5 MHz, $CDCl_3$):	165.27, 148.84, 146.15, 143.29, 140.48, 137.12, 130.63, 129.32, 129.04, 128.04, 127.12, 126.92, 121.32, 35.37, 35.31, 33.56, 31.45, 29.46, 29.44, 29.23, 22.36, 13.95.
FT-IR (vmax, cm^{-1}):	692, 758, 779, 821, 860, 883, 945, 1004, 1016, 1082, 1163, 1190, 1269 (C-O stretching), 1398, 1463 (aliphatic C-H bending), 1506 (aromatic C-C stretching), 1604, 1722 (C=O stretching), 2846 (aliphatic C-H stretching), 2918 (aliphatic C-H stretching).
MS (ESI+, m/z):	807.4383 (100%, $C_{55}H_{60}NaO_4$, calcd. for $C_{55}H_{60}NaO_4$ 807.4384, M + Na)

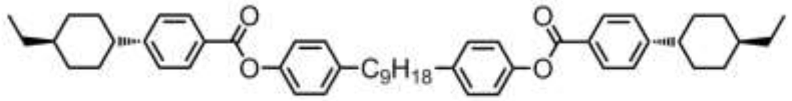

Nonane-1,9-diyl*bis*(4,1-phenylene) *bis*(4-(*trans* 4-ethylcyclohexyl)benzoate), 5

Yield:	585 mg (79 %)
^{1}H NMR (400 MHz, $CDCl_3$):	0.91 (6H, t, *J* = 7.2 Hz, <u>CH3</u>-CH2-CH2-Cy), 0.96 – 1.16 (4H, m, Cy<u>H</u>), 1.16 – 1.40 (16H, m, Ar-CH2-(<u>CH2</u>)7-CH2-Ar + CyH), 1.47 (4H, dquart, *J* = 2.8 Hz, *J* = 12.0 Hz, Cy<u>H</u>), 1.40 – 1.80 (4H, m, CH3-<u>CH2</u>-Cy),1.86 – 1.94 (8H, m, Cy<u>H</u>), 2.54 (2H, tt, *J* =2.8 Hz, *J* = 12.0 Hz, Cy<u>H</u>), 2.60 (4H, t, *J* = 7.6 Hz, Ar-<u>CH2</u>-(CH2)7-<u>CH2</u>-Ar), 7.07 (4H, ddd, *J* = 2.1 Hz, *J* = 2.5 Hz, *J* = 8.2 Hz, Ar<u>H</u>), 7.20 (4H, ddd, *J* = 2.1 Hz, *J* = 2.5 Hz, *J* = 8.2 Hz, Ar<u>H</u>), 7.33 (4H, ddd, *J* = 2.1 Hz, *J* = 2.5 Hz, *J* = 8.2 Hz, Ar<u>H</u>), 8.10 (4H, ddd, *J* = 2.1 Hz, *J* = 2.5 Hz, *J* = 8.2 Hz, Ar<u>H</u>)
^{13}C NMR (100.5 MHz, $CDCl_3$):	165.38, 154.02, 148.86, 140.38, 130.26, 129.28, 127.23, 127.05, 121.33, 44.81, 38.97, 35.36, 33.99, 32.97, 31.46, 29.91, 29.47, 29.45, 29.23, 11.50.
FT-IR (vmax, cm^{-1}):	692, 721, 758, 779, 821, 883, 945, 1004, 1016, 1073, 1111, 1165, 1199, 1269 (C-O stretching), 1373, 1398, 1463 (aliphatic C-H bending), 1506 (Aromatic C-C stretching), 1604, 1722 (C=O stretching), 2846 (aliphatic C-H stretching), 2918 (aliphatic C-H stretching).
MS (ESI+, m/z):	791.5062 ($C_{53}H_{68}NaO_4$, calcd. for $C_{53}H_{68}NaO_4$ 791.5010, M + Na)

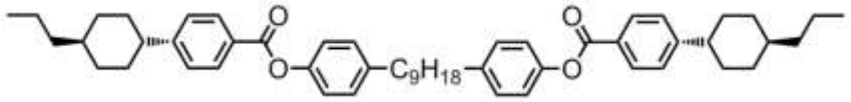

Nonane-1,9-diyl*bis*(4,1-phenylene) *bis*(4-(*trans* 4-propylcyclohexyl)benzoate), 6

Yield:	552 mg (72 %)
^{1}H NMR (400 MHz, $CDCl_3$):	0.89 (6H, t, *J* = 7.0 Hz, CH_3-CH_2-CH_2-Cy), 0.99 – 1.12 (4H, m, CyH), 1.17 – 1.37 (20H, m, Ar-CH_2-$(CH_2)_7$-CH_2-Ar + CyH), 1.47 (4H, dquart, *J* = 3.1 Hz, *J* = 12.5 Hz, CyH), 1.55 – 1.63 (4H, quintet, *J* = 7.0 Hz, CH_3-CH_2-CH_2-Cy),1.84 – 1.94 (8H, m, CyH), 2.54 (2H, tt, *J* =3.1 Hz, *J* = 12.2 Hz, CyH), 2.60 (4H, t, *J* = 7.0 Hz, Ar-CH_2-$(CH_2)_7$-CH_2-Ar), 7.08 (4H, ddd, *J* = 1.8 Hz, *J* = 2.8 Hz, *J* = 8.2 Hz, ArH), 7.20 (4H, ddd, *J* = 1.8 Hz, *J* = 2.8 Hz, *J* = 8.2 Hz, ArH), 7.32 (4H, ddd, *J* = 1.8 Hz, *J* = 2.5 Hz, *J* = 8.5 Hz, ArH), 8.10 (4H, ddd, *J* = 1.8 Hz, *J* = 2.5 Hz, *J* = 8.5 Hz, ArH)
^{13}C NMR (100.5 MHz, $CDCl_3$):	165.38, 154.03, 148.86, 140.38, 130.26, 129.28, 127.23, 127.05, 121.34, 44.81, 39.63, 36.92, 35.36, 34.02, 33.37, 31.46, 29.47, 29.44, 29.23, 19.99, 14.39.
FT-IR (vmax, cm^{-1}):	698, 721, 759, 823, 883, 1016, 1074, 1165, 1178, 1197, 1267 (C-O stretching), 1398, 1463 (aliphatic C-H bending), 1506 (Aromatic C-C stretching), 1606, 1722 (C=O stretching), 2846 (aliphatic C-H stretching), 2918 (aliphatic C-H stretching).
MS (ESI+, m/z):	763.4672 (100%, $C_{51}H_{64}NaO_4$, calcd. for $C_{51}H_{64}NaO_4$ 763.4697, M + Na)

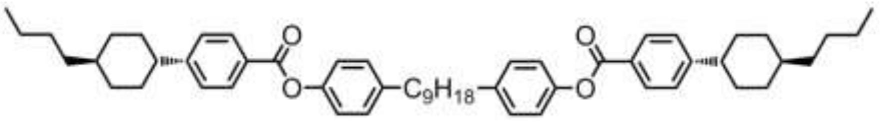

Nonane-1,9-diyl*bis*(4,1-phenylene) *bis*(4-(*trans* 4-propylcyclohexyl)benzoate), 7

Yield:	693 mg (80 %)
^{1}H NMR (400 MHz, $CDCl_3$):	0.82 (6H, t, *J* = 7.6 Hz, CH_3-CH_2-CH_2-CH_2-Cy), 0.94 – 1.07 (4H, m, CyH), 1.12 – 1.31 (24H, m, Ar-CH_2-$(CH_2)_7$-CH_2-Ar + CyH), 1.40 (4H, dquart, *J* = 2.8 Hz, *J* = 12.5 Hz, CyH), 1.54 (4H, quintet, *J* = 7.6 Hz, CH_3-CH_2-CH_2-CH_2-Cy),1.77 – 1.87 (8H, m, CyH), 2.48 (2H, tt, *J* =2.5 Hz, *J* = 12.5 Hz, CyH), 2.54 (4H, t, *J* = 7.0 Hz, Ar-CH_2-$(CH_2)_7$-CH_2-Ar), 7.02 (4H, ddd, *J* = 2.1nHz, *J* = 2.4 Hz, *J* = 8.5 Hz, ArH), 7.14 (4H, ddd, *J* = 2.1 Hz, *J* = 2.4 Hz, *J* = 8.5 Hz, ArH), 7.26 (4H, d, *J* = 8.2 Hz, ArH), 8.03 (4H, d, *J* = 8.2 Hz, ArH)
^{13}C NMR (100.5 MHz, $CDCl_3$):	165.39, 154.04, 148.86, 140.39, 130.26, 129.29, 127.23, 127.05, 121.34, 44.82, 37.20, 37.04, 35.36, 34.03, 33.41, 31.46, 29.47, 29.45, 29.23, 29.20, 22.99, 14.16.
FT-IR (vmax, cm^{-1}):	698, 721, 759, 823, 1016, 1074, 1111, 1165, 1178, 1217, 1228, 1265 (C-O stretching), 1365, 1461 (aliphatic C-H bending), 1506 (Aromatic C-C stretching), 1606, 1723 (C=O stretching), 2846 (aliphatic C-H stretching), 2918 (aliphatic C-H stretching).
MS (ESI+, m/z):	819.5332 (100%, $C_{55}H_{72}NaO_4$, cacld. for $C_{55}H_{72}NaO_4$ 819.5323, M + Na)

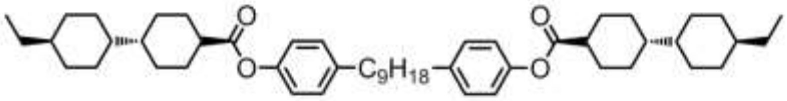

Nonane-1,9-diylbis(4,1-phenylene) *bis*(*trans trans* 4'-ethyl-[1,1'-bi(cyclohexane)]-4-carboxylate), 9

Yield:	496 mg (66 %)
^{1}H NMR (400 MHz, $CDCl_3$):	0.71 – 1.29 (38H, m, Ar-CH_2($\underline{CH_2}$)$_7$-CH_2-Ar + Cy$\underline{H}$ + $\underline{CH_3}$-CH_2-Cy), 1.39 – 1.55 (8H, m, Cy$\underline{H}$), 1.61 – 1.73 (8H, m, Cy$\underline{H}$), 1.74 – 1.81 (4H, m, Cy$\underline{H}$), 2.03 – 2.11 (4H, m, Cy$\underline{H}$), 2.36 (2H, tt, *J* = 3.7 Hz, *J* = 12.2 Hz, Cy$\underline{H}$), 2.50 (4H, t, *J* = 7.6 Hz, Ar-$\underline{CH_2}$-(CH_2)$_7$-$\underline{CH_2}$-Ar), 6.87 (4H, ddd, *J* = 1.8 Hz, *J* = 2.5 Hz, *J* = 8.2 Hz, Ar$\underline{H}$), 7.08 (4H, ddd, *J* = 1.8 Hz, *J* = 2.5 Hz, *J* = 8.2 Hz, Ar$\underline{H}$)
^{13}C NMR (100.5 MHz, $CDCl_3$):	174.91, 148.70, 140.20, 129.17, 121.13, 43.67, 43.22, 42.48, 39.63, 35.31, 33.10, 31.43, 30.00, 29.96, 29.44, 29.42, 29.28, 29.20, 29.13, 11.53.
FT-IR (vmax, cm^{-1}):	501, 624, 725, 840, 987, 1126, 1195, 1249 (C-O stretching), 1319, 1373, 1450 (aliphatic C-H bending), 1597, 1689, 1743 (C=O stretching), 2669, 2846 (aliphatic C-H stretching), 2916 (aliphatic C-H stretching).
MS (ESI+, m/z):	775.5664 (100%, $C_{51}H_{76}NaO_4$, calcd. for $C_{51}H_{76}NaO_4$ 775.5636, M + Na)

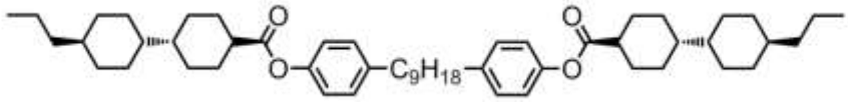

Nonane-1,9-diyl*bis*(4,1-phenylene) *bis*(*trans trans* 4'-propyl-[1,1'-bi(cyclohexane)]-4-carboxylate), 10

Yield:	537 mg (71 %)
^{1}H NMR (400 MHz, $CDCl_3$):	0.73 – 1.27 (42H, m, Ar-CH_2($\underline{CH_2}$)$_7$-CH_2-Ar + Cy$\underline{H}$ + $\underline{CH_3}$-CH_2- CH_2-Cy), 1.39 – 1.56 (8H, m, Cy$\underline{H}$), 1.62 – 1.82 (12H, m, Cy$\underline{H}$), 2.04 – 2.11 (4H, m, Cy$\underline{H}$), 2.36 (2H, tt, *J* = 3.7 Hz, *J* = 12.2 Hz, Cy$\underline{H}$), 2.50 (4H, t, *J* = 7.3 Hz, Ar-$\underline{CH_2}$-(CH_2)$_7$-$\underline{CH_2}$-Ar), 6.87 (4H, ddd, *J* = 1.8 Hz, *J* = 2.8 Hz, *J* = 8.5 Hz, Ar$\underline{H}$), 7.08 (4H, ddd, *J* = 1.8 Hz, *J* = 2.8 Hz, *J* = 8.5 Hz, Ar$\underline{H}$)
^{13}C NMR (100.5 MHz, $CDCl_3$):	43.68, 43.22, 42.49, 39.77, 37.57, 35.31, 33.51, 31.43, 29.98, 29.45, 29.42, 29.28, 29.20, 29.13, 20.03, 14.41.
FT-IR (vmax, cm^{-1}):	516, 725, 771, 840, 987, 1080, 1126, 1165, 1195, 1273 (C-O stretching), 1373, 1450 (aliphatic C-H bending),, 1504, 1604, 1735 (C=O stretching), 2846 (aliphatic C-H stretching), 2916 (aliphatic C-H stretching).
MS (ESI+, m/z):	803.5958 (100%, $C_{53}H_{80}NaO_4$, calcd. for $C_{53}H_{80}NaO_4$ 803.5949, M + Na)

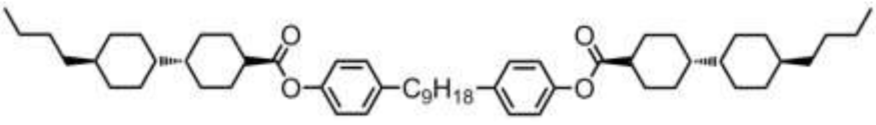

Nonane-1,9-diyl*bis*(4,1-phenylene) *bis*(*trans trans* 4'-butyl-[1,1'-bi(cyclohexane)]-4-carboxylate), 11

Yield: 533 mg (68 %)

^{1}H NMR (400 MHz, $CDCl_3$): 0.76 – 1.34 (46H, m, Ar-$CH_2(CH_2)_7$-CH_2-Ar + CyH + CH_3-CH_2-CH_2-CH_2-Cy), 1.44 – 1.61 (8H, m, CyH), 1.66 – 1.78 (8H, m, CyH), 1.79 – 1.85 (4H, m, CyH), 2.09 – 2.17 (4H, m, CyH), 2.41 (2H, tt, *J* = 3.4 Hz, *J* = 12.2 Hz, CyH), 2.55 (4H, t, *J* = 7.3 Hz, Ar-CH_2-$(CH_2)_7$-CH_2-Ar), 6.92 (4H, d, *J* = 8.2 Hz, ArH), 7.13 (4H, d, *J* = 8.2 Hz, ArH)

^{13}C NMR (100.5 MHz, $CDCl_3$): 174.90, 148.70, 140.19, 129.16, 121.12, 43.67, 43.21, 42.48, 37.84, 37.16, 35.30, 33.54, 31.42, 29.98, 29.43, 29.41, 29.27, 29.24, 29.19, 29.12, 23.01, 14.15

FT-IR (vmax, cm^{-1}): 516, 632, 694, 763, 840, 979, 1072, 1126, 1165, 1203 (C-O stretching), 1373, 1450 (aliphatic C-H bending), 1504, 1604, 1743 (C=O stretching), 2846 (aliphatic C-H stretching), 2916 (aliphatic C-H stretching).

MS (ESI+, m/z): 831.6275 (100%, $C_{55}H_{84}NaO_4$, calcd. for $C_{53}H_{80}NaO_4$ 831.6262, M + Na)

1.4. ^{1}H and ^{13}C NMR Spectra

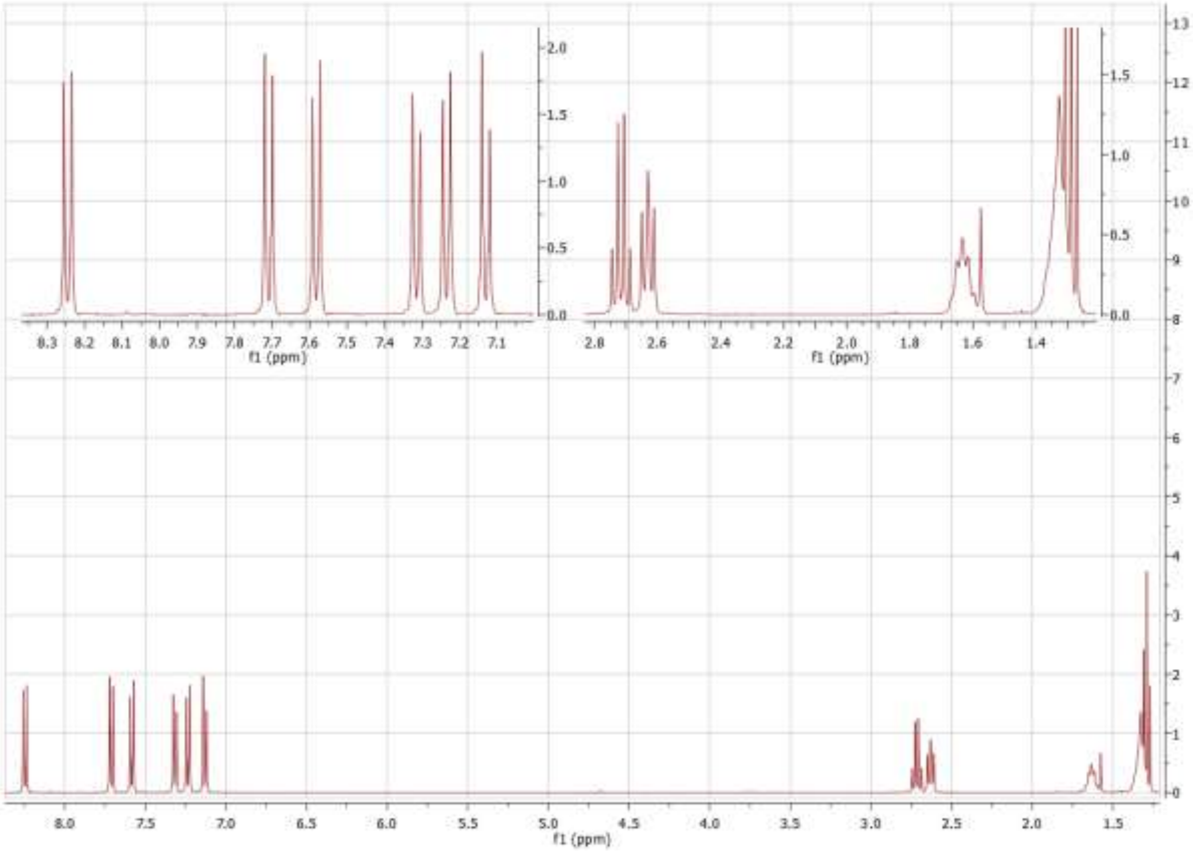

Figure SI2: ^{1}H NMR spectra (400 MHz, $CDCl_3$) of compound **1**, with expansions of the aromatic (top left) and aliphatic regions (top right)

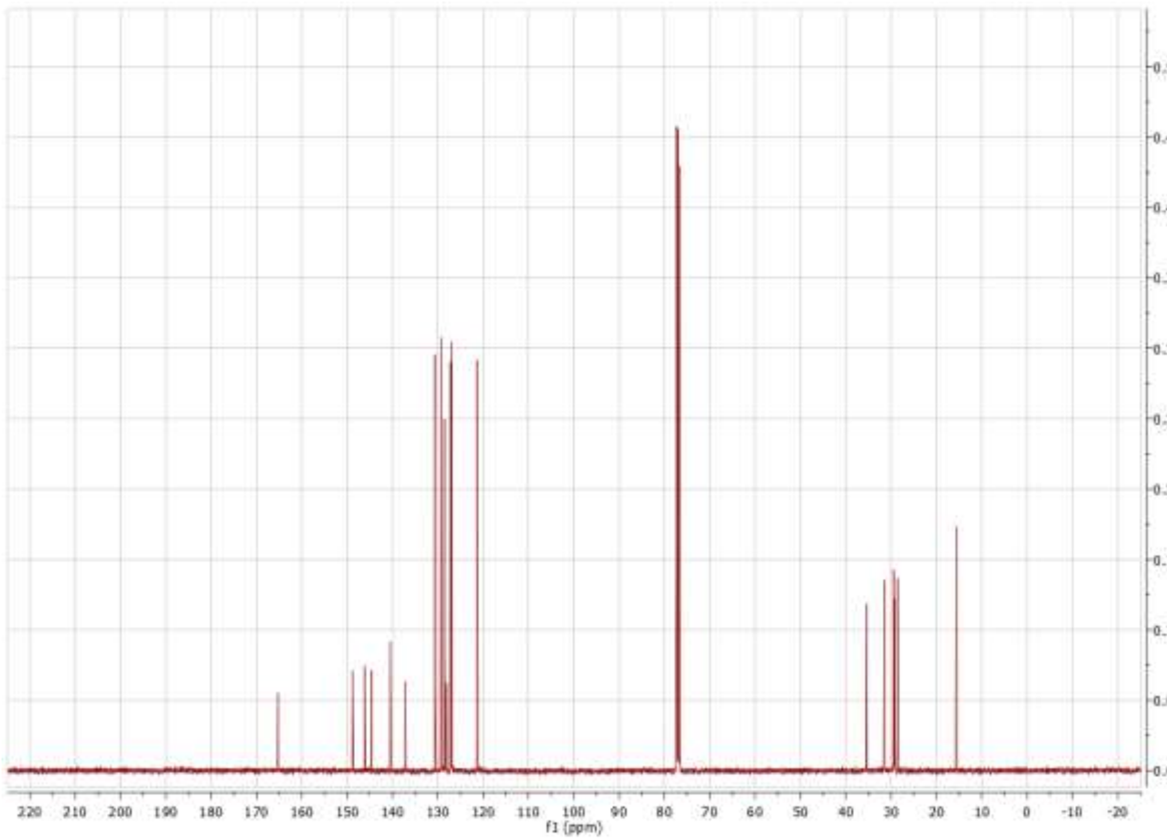

Figure SI3: ^{13}C NMR spectra (100.5 MHz, $CDCl_3$) of compound **1**

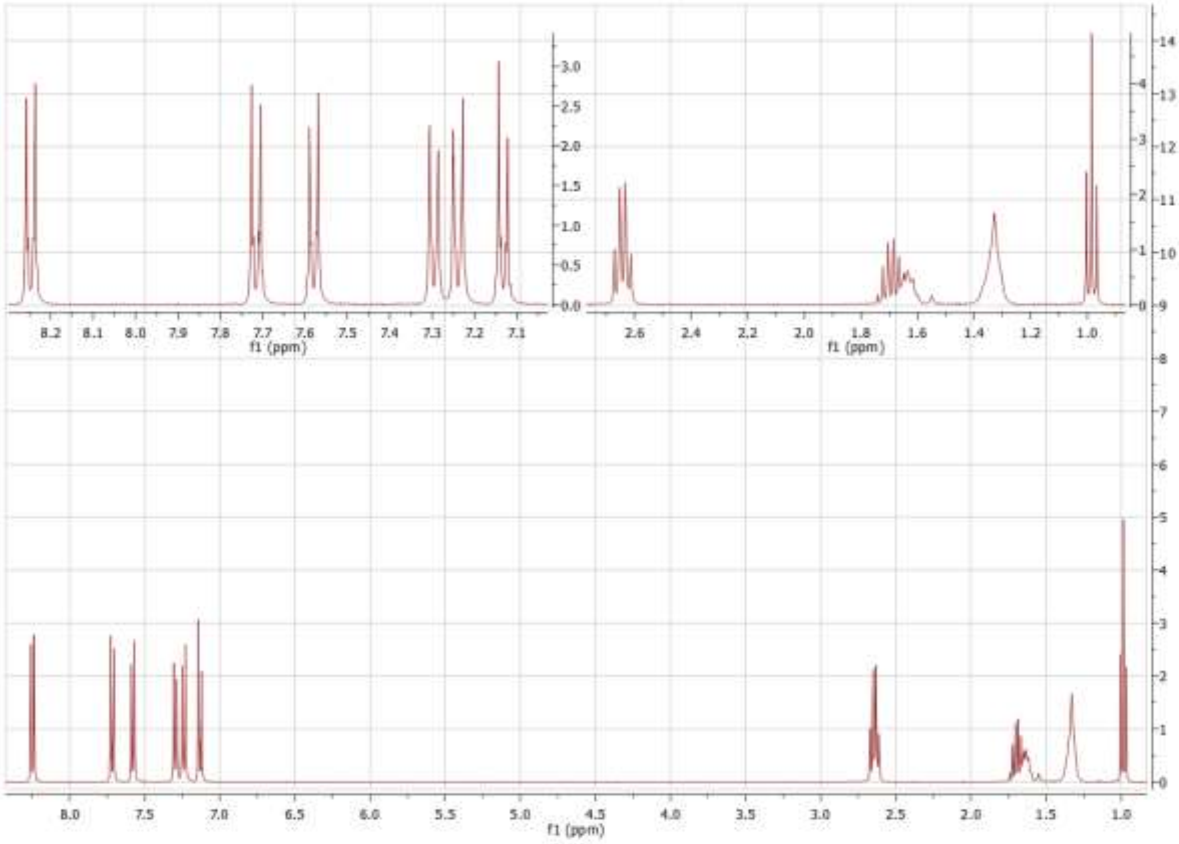

Figure SI4: ^{1}H NMR spectra (400 MHz, $CDCl_3$) of compound **2**, with expansions of the aromatic (top left) and aliphatic regions (top right)

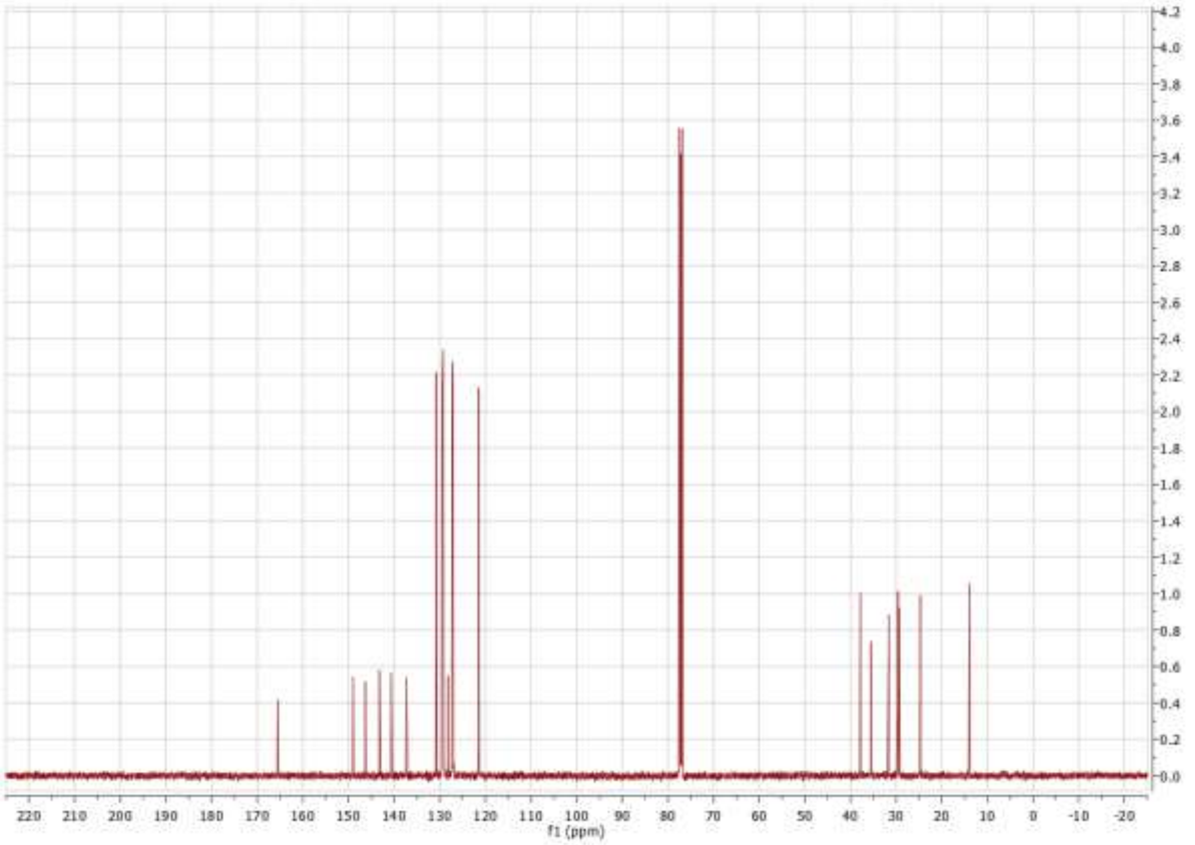

Figure SI5: ^{13}C NMR spectra (100.5 MHz, $CDCl_3$) of compound **2**

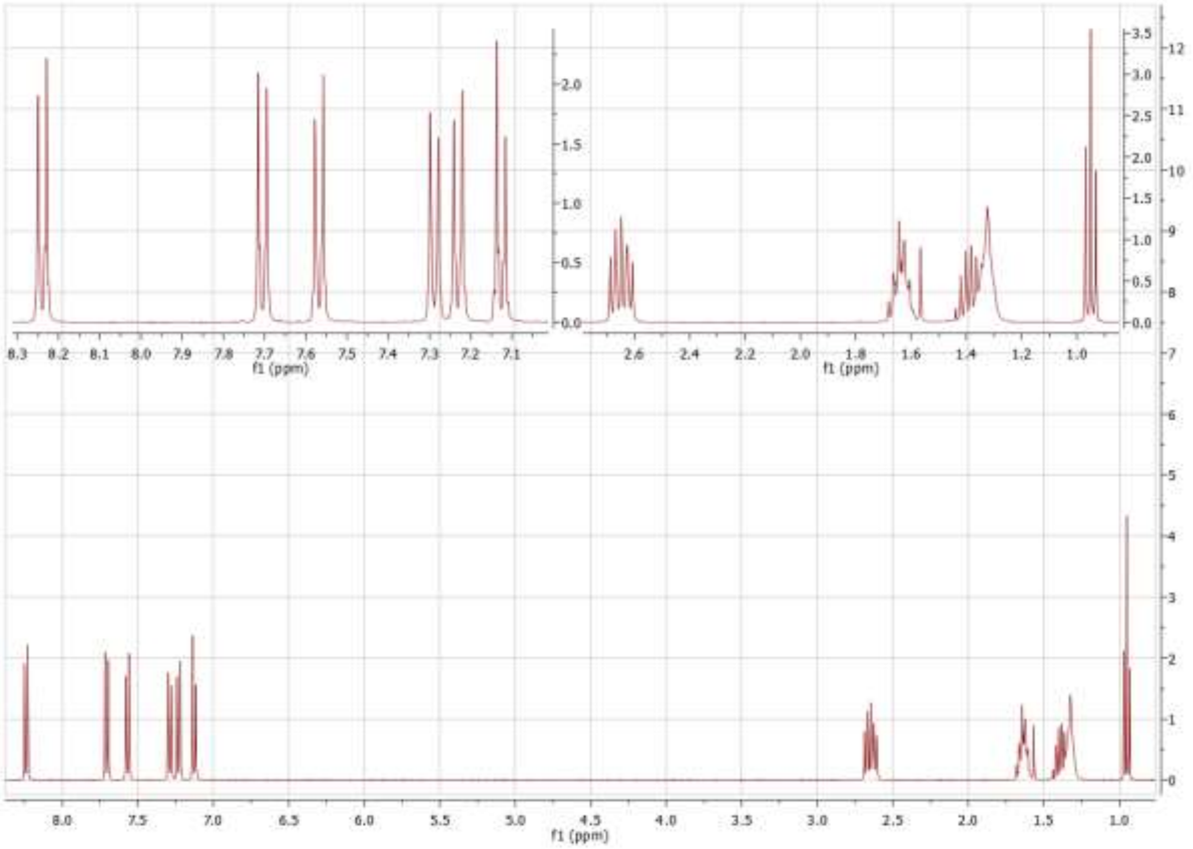

Figure SI6: ^1H NMR spectra (400 MHz, $CDCl_3$) of compound **3**, with expansions of the aromatic (top left) and aliphatic regions (top right)

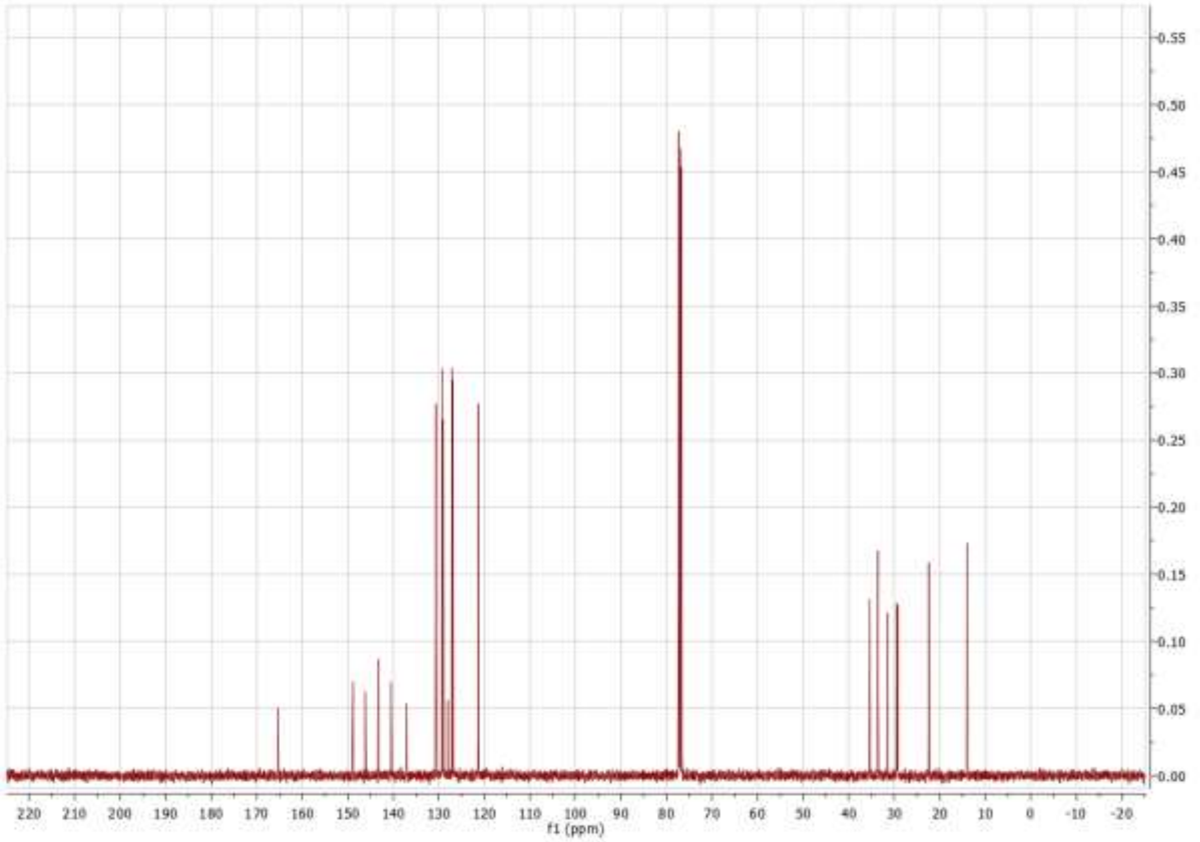

Figure SI7: ^{13}C NMR spectra (100.5 MHz, $CDCl_3$) of compound **3**

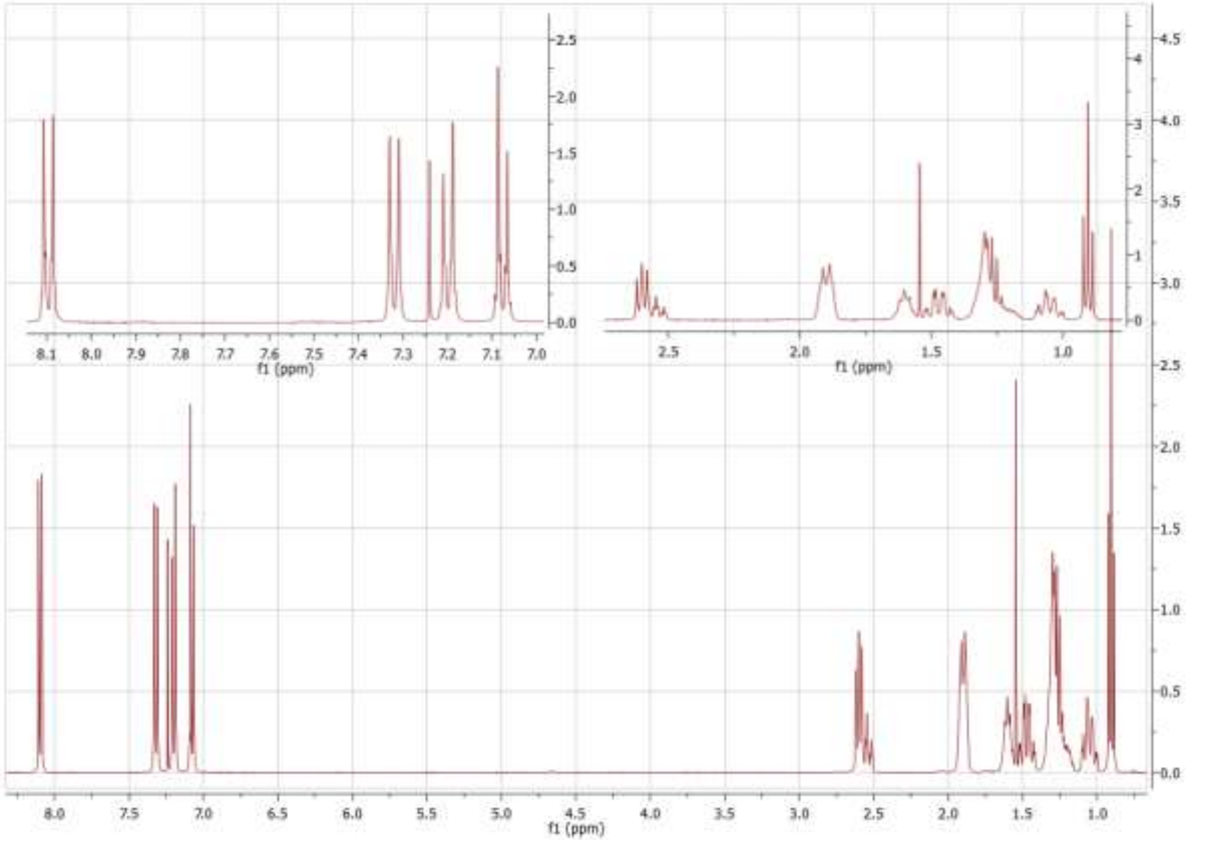

Figure SI8: ^1H NMR spectra (400 MHz, $CDCl_3$) of compound **5**, with expansions of the aromatic (top left) and aliphatic regions (top right)

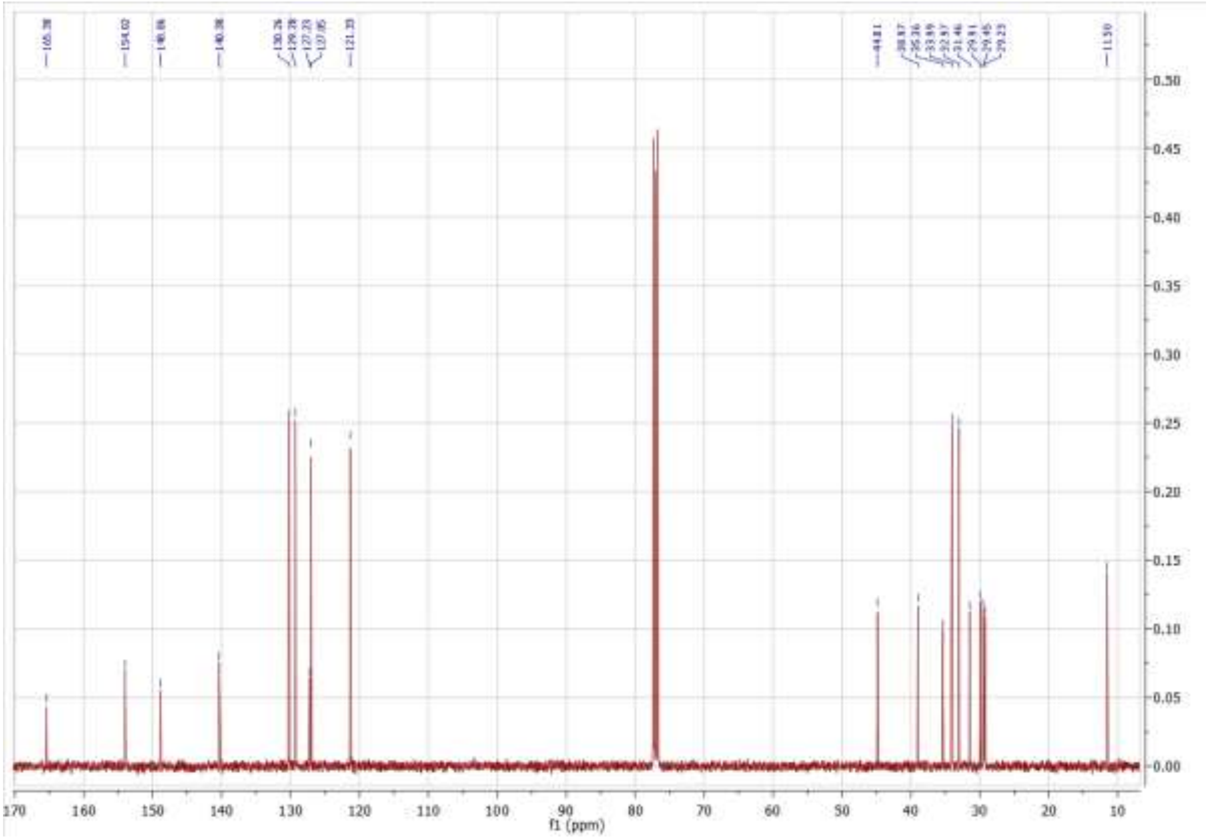

Figure SI9: ^{13}C NMR spectra (100.5 MHz, $CDCl_3$) of compound **5**

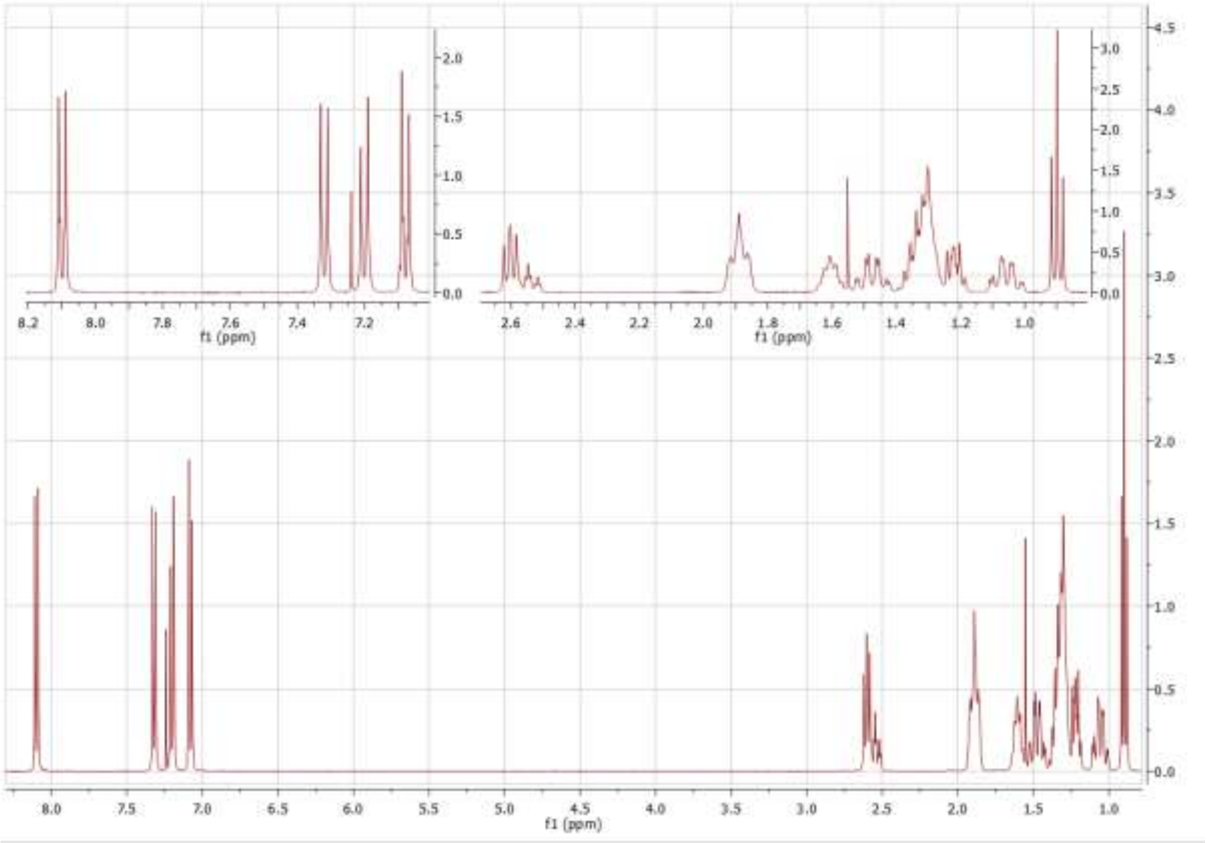

Figure SI10: ^{1}H NMR spectra (400 MHz, $CDCl_3$) of compound **6**, with expansions of the aromatic (top left) and aliphatic regions (top right)

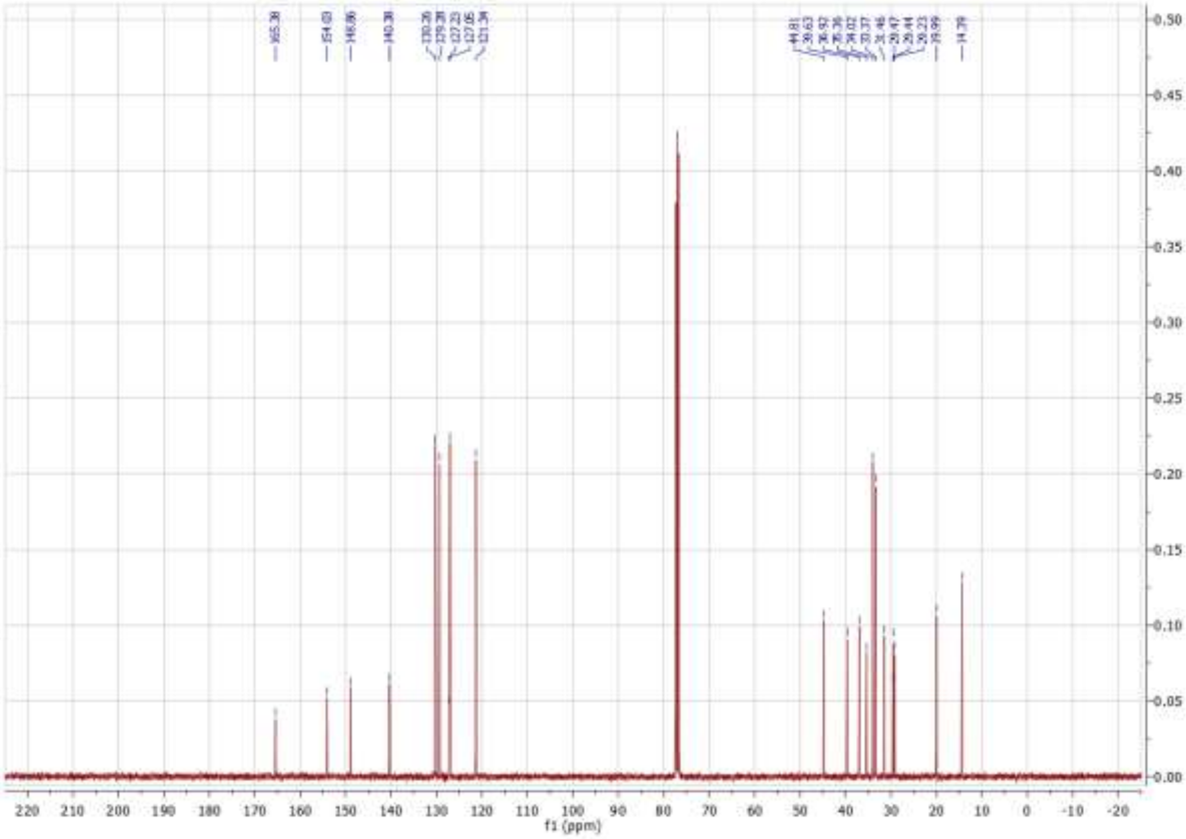

Figure SI11: ^{13}C NMR spectrum (100.5 MHz, $CDCl_3$) of compound **6**

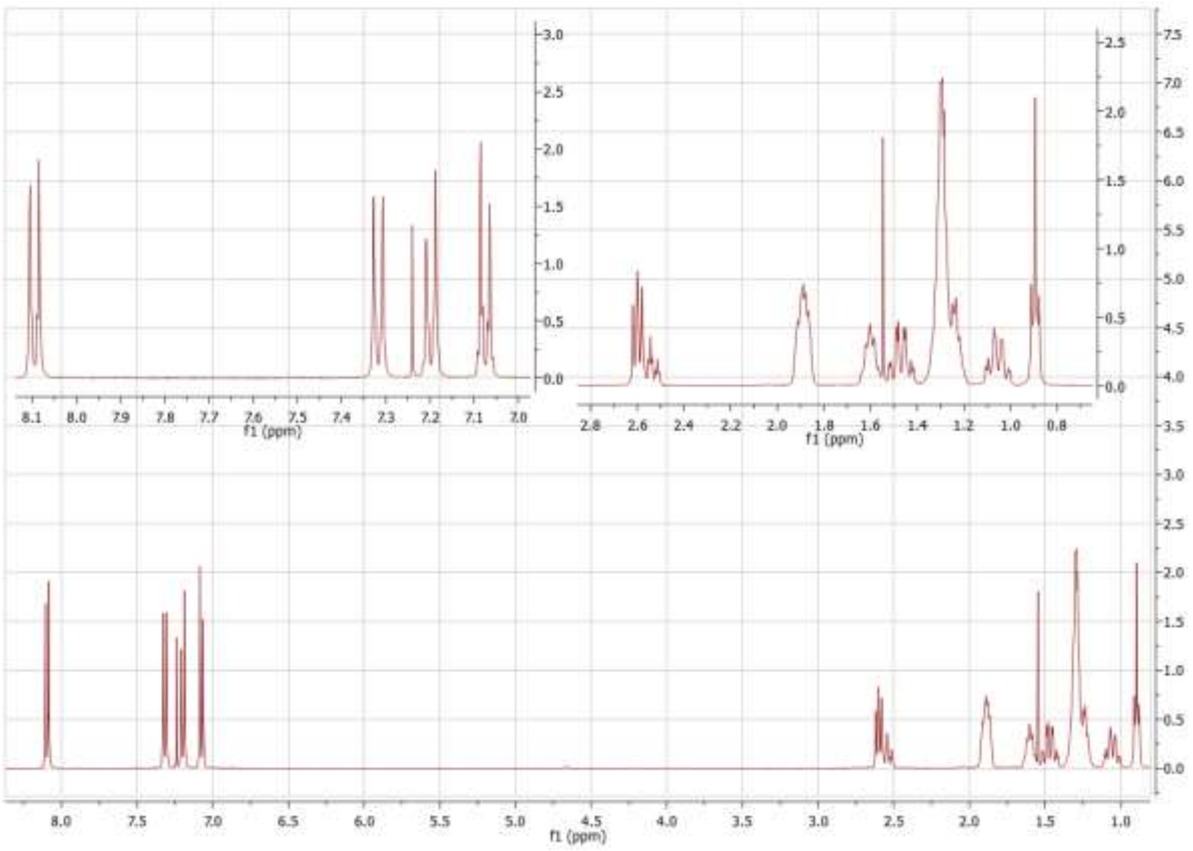

Figure SI12: ^{1}H NMR spectra (400 MHz, $CDCl_3$) of compound **7**, with expansions of the aromatic (top left) and aliphatic regions (top right)

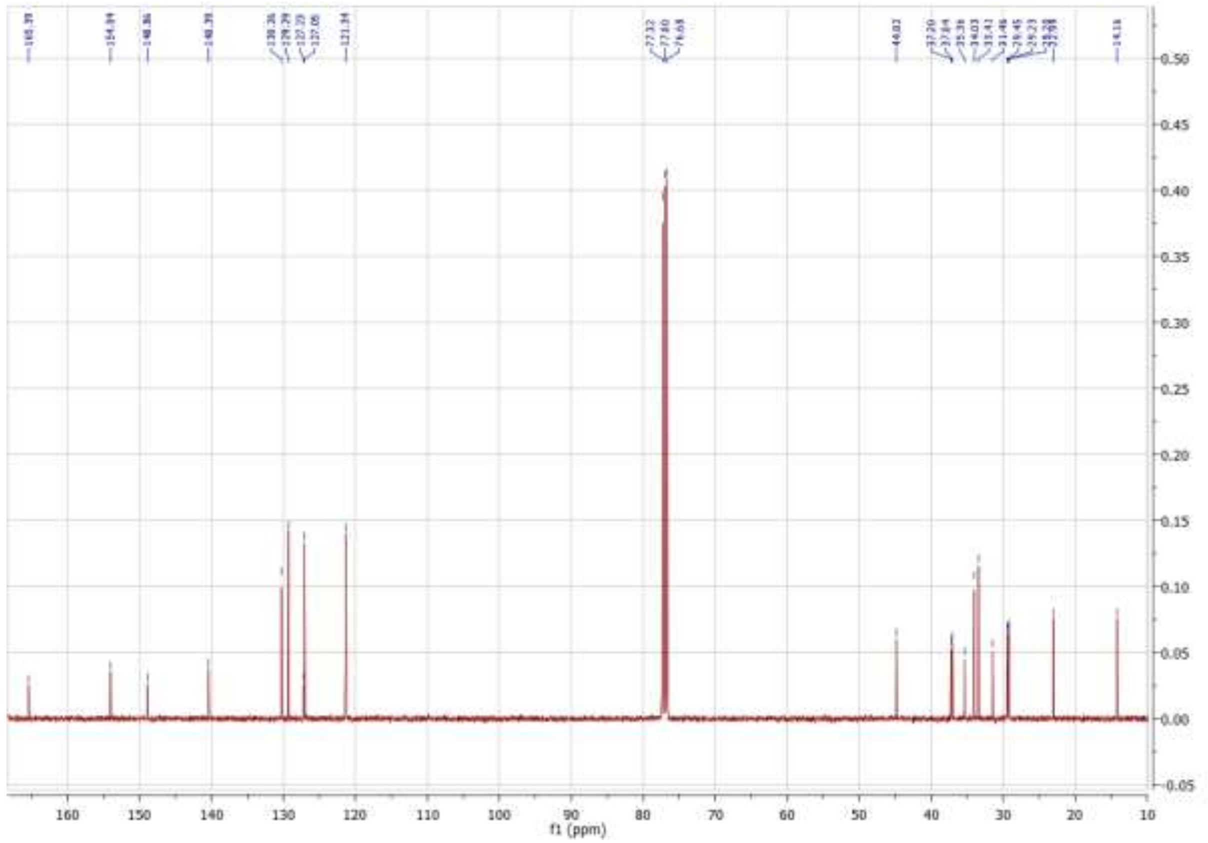

Figure SI13: ^{13}C NMR spectrum (100.5 MHz, $CDCl_3$) of compound **7**

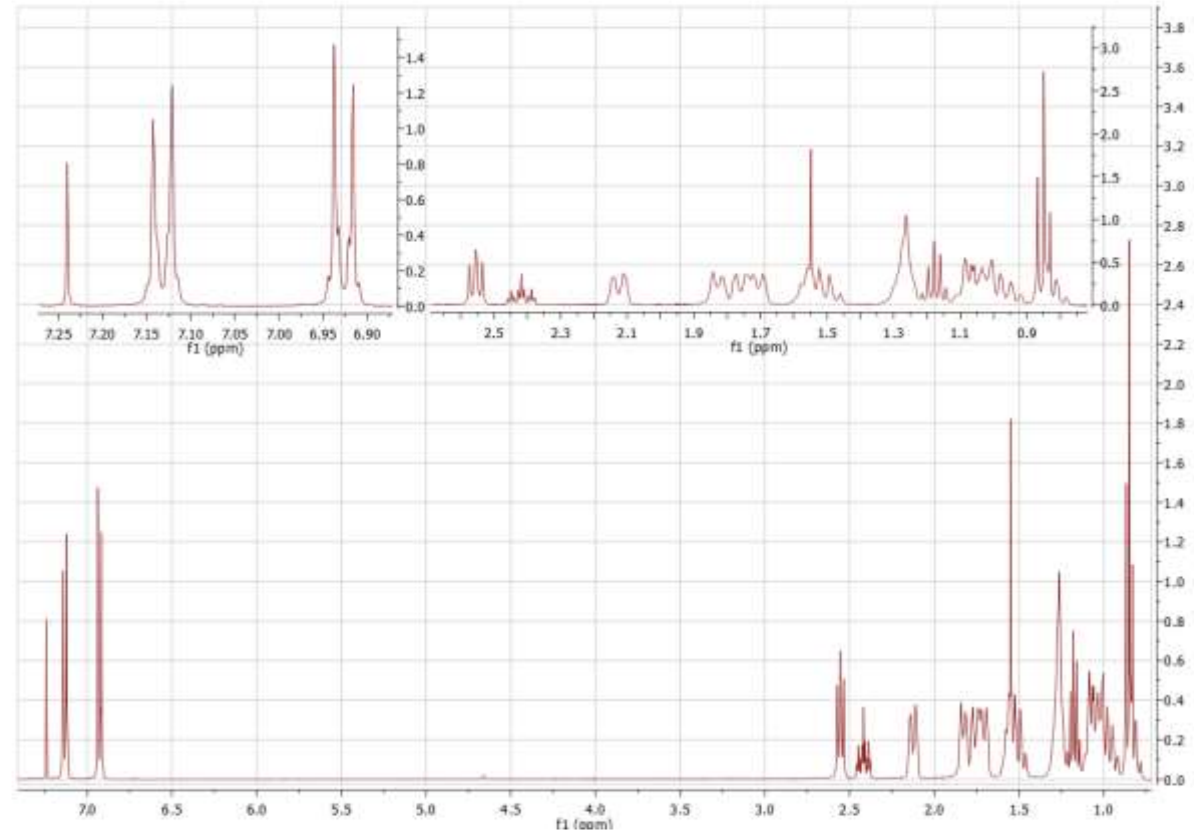

Figure SI14: ^1H NMR spectra (400 MHz, $CDCl_3$) of compound **9**, with inserts showing the aromatic (top left) and aliphatic regions (top right)

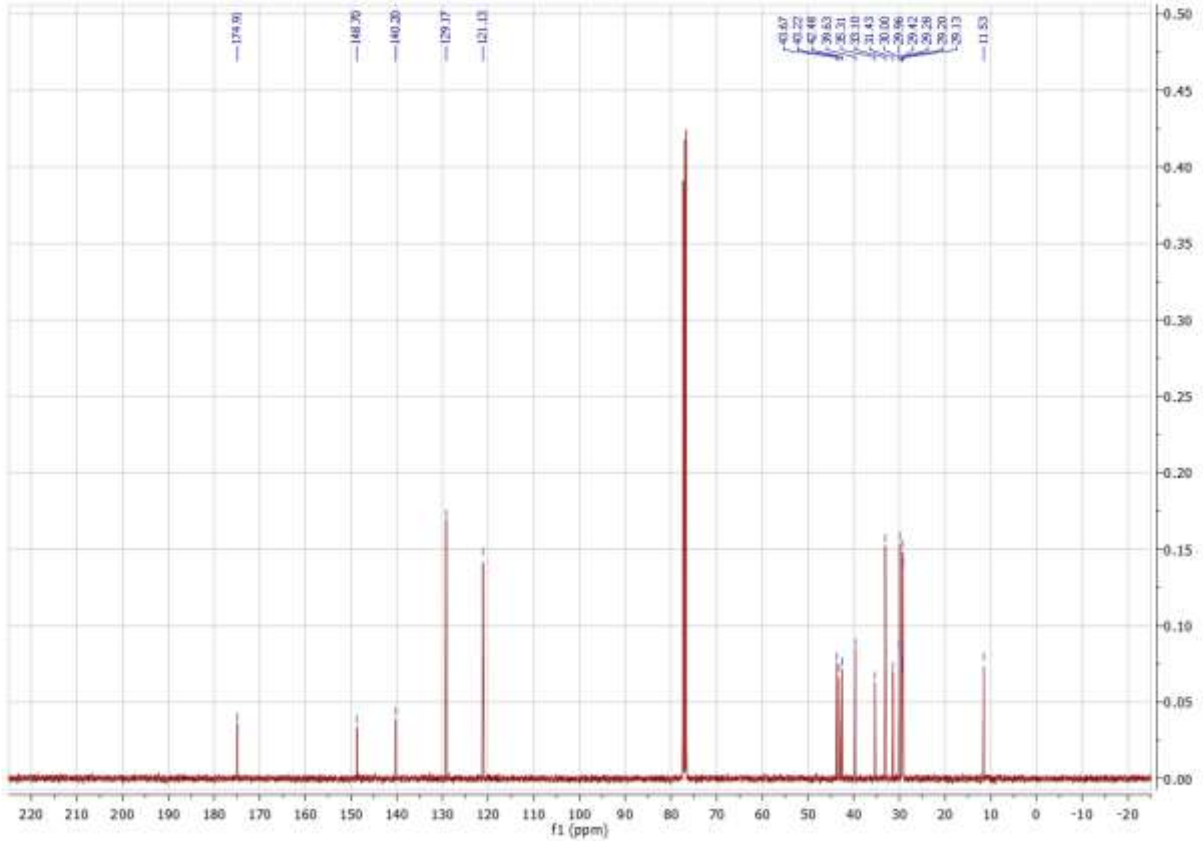

Figure SI15: ^{13}C NMR spectra (100.5 MHz, $CDCl_3$) of compound **9**

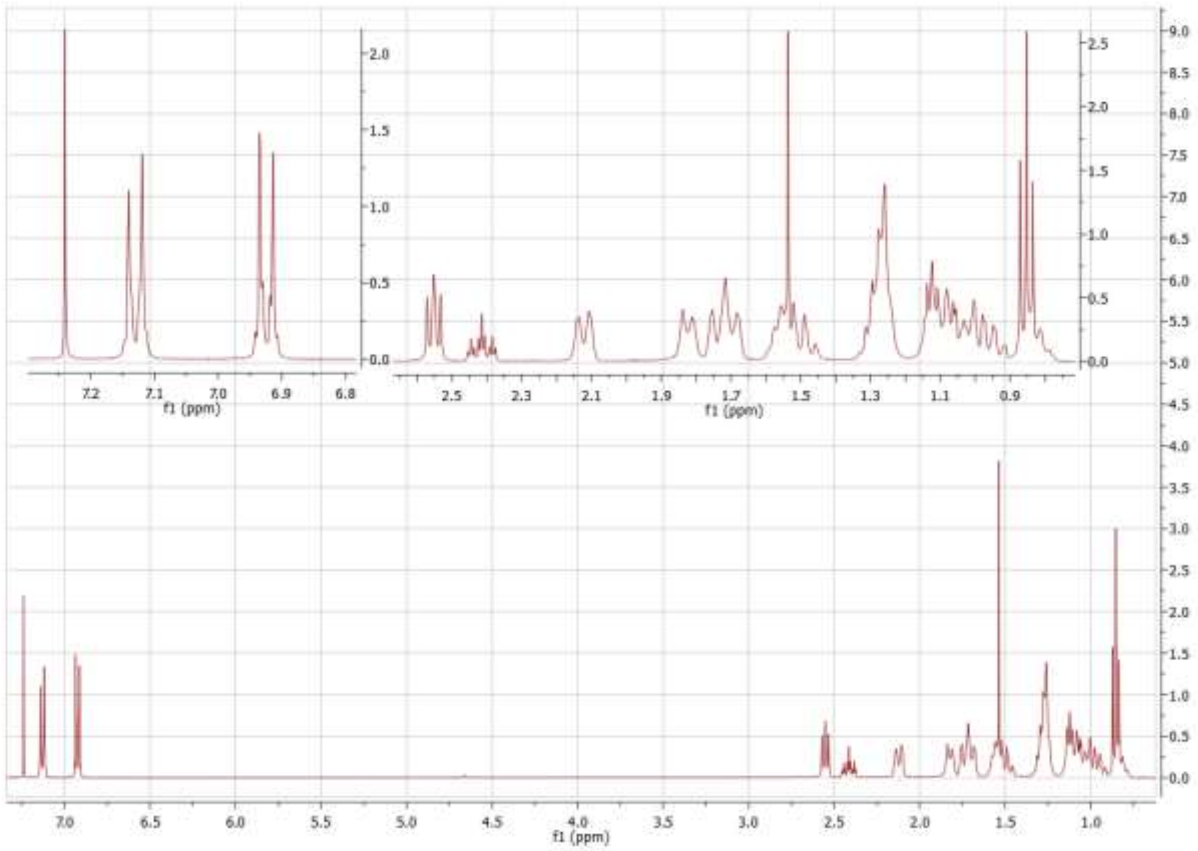

Figure SI16: ^1H NMR spectra (400 MHz, $CDCl_3$) of compound **10**, with inserts showing the aromatic (top left) and aliphatic regions (top right)

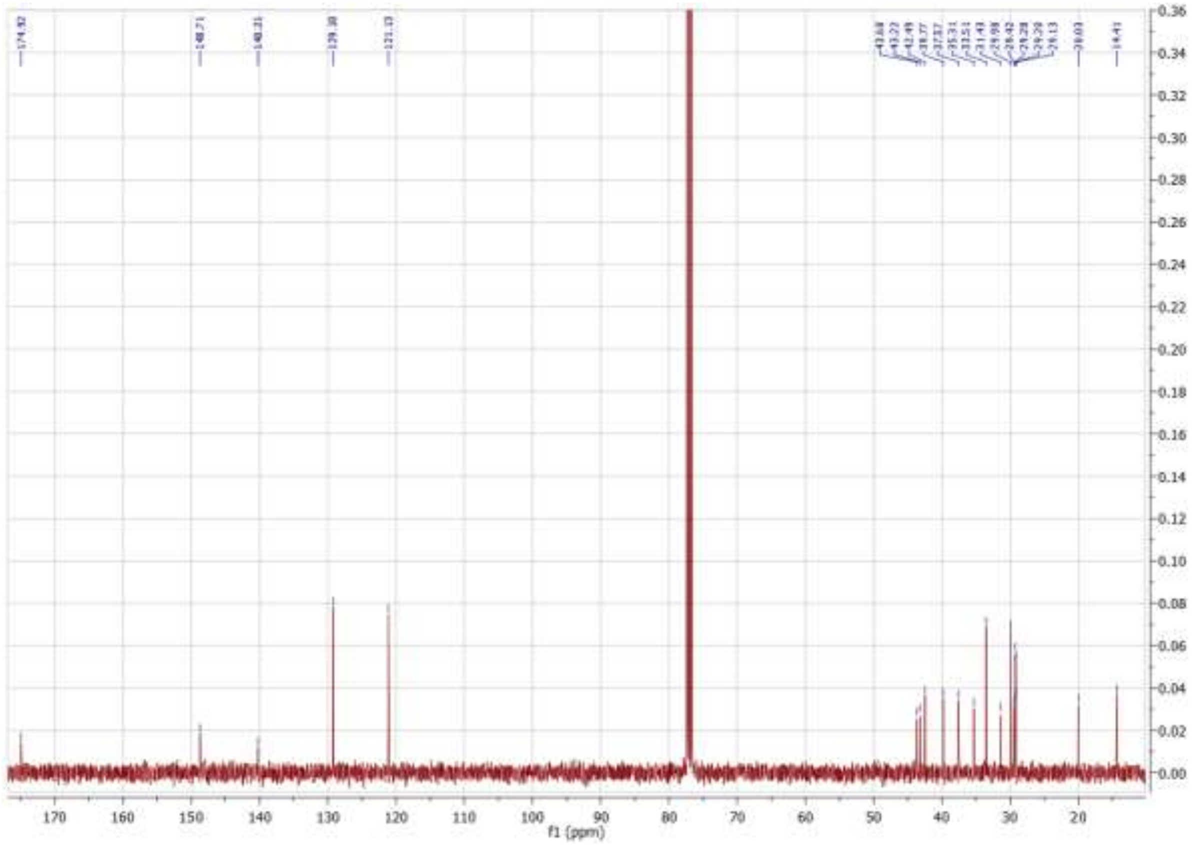

Figure SI17: ^{13}C NMR spectra (100.5 MHz, $CDCl_3$) of compound **10**

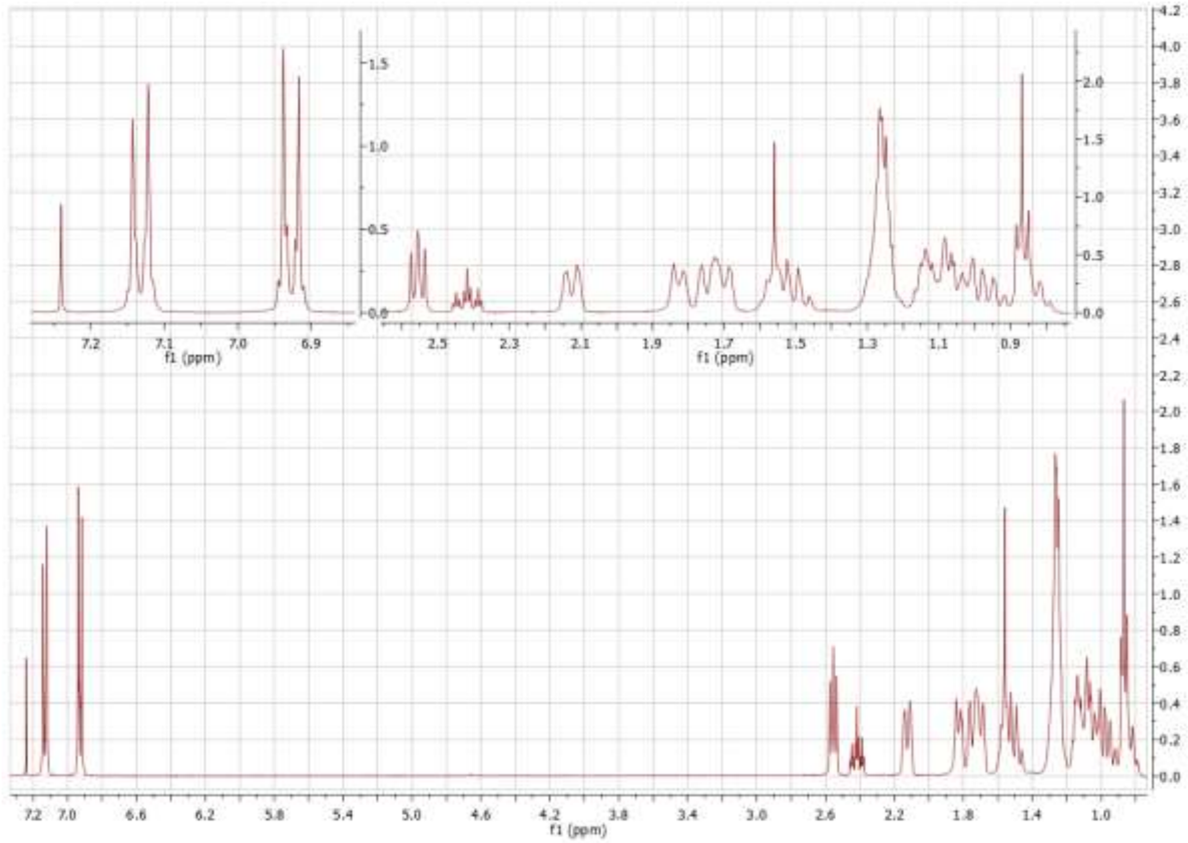

Figure SI18: ^{1}H NMR spectra (400 MHz, $CDCl_3$) of compound **11**, with inserts showing the aromatic (top left) and aliphatic regions (top right)

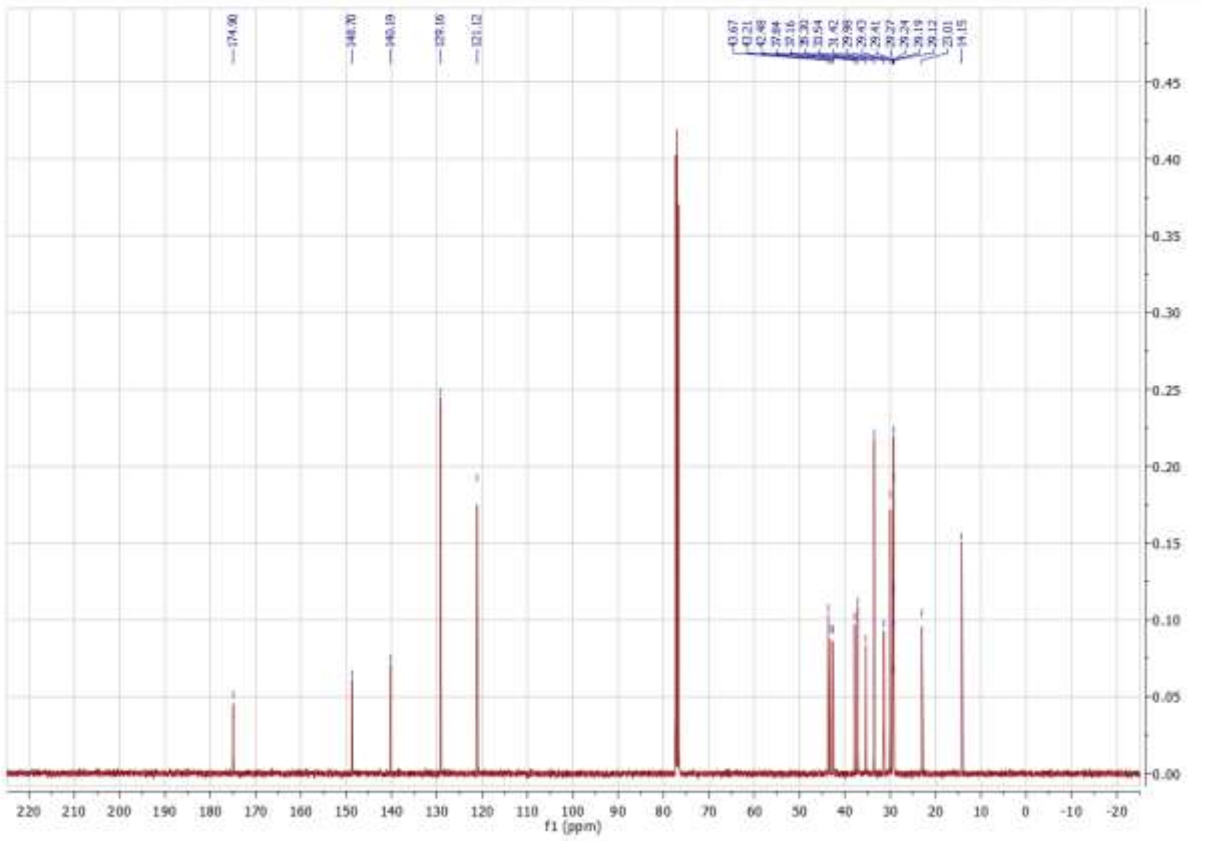

Figure SI19: ^{13}C NMR spectra (100.5 MHz, $CDCl_3$) of compound **11**

1.5. Differential Scanning Calorimetry Traces

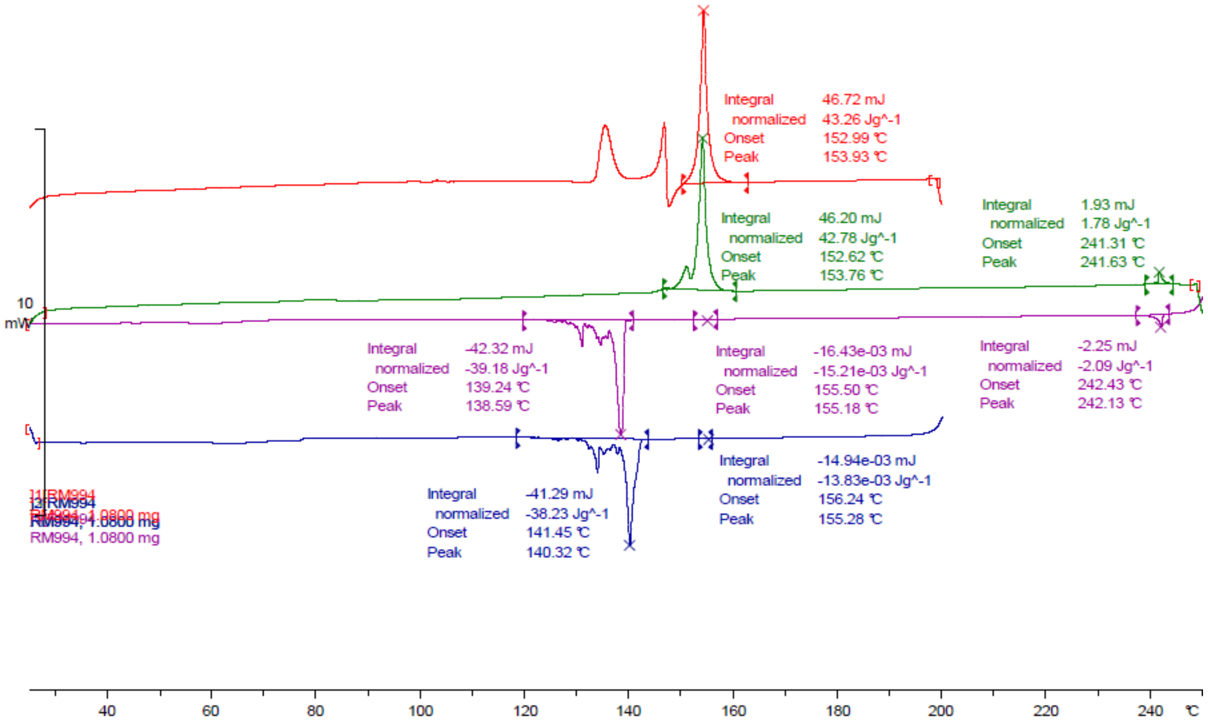

Figure SI20: DSC trace of compound **1** obtained at a heat/cool rate of 10 °C min^{-1} and a sample weight of 1.08 mg.

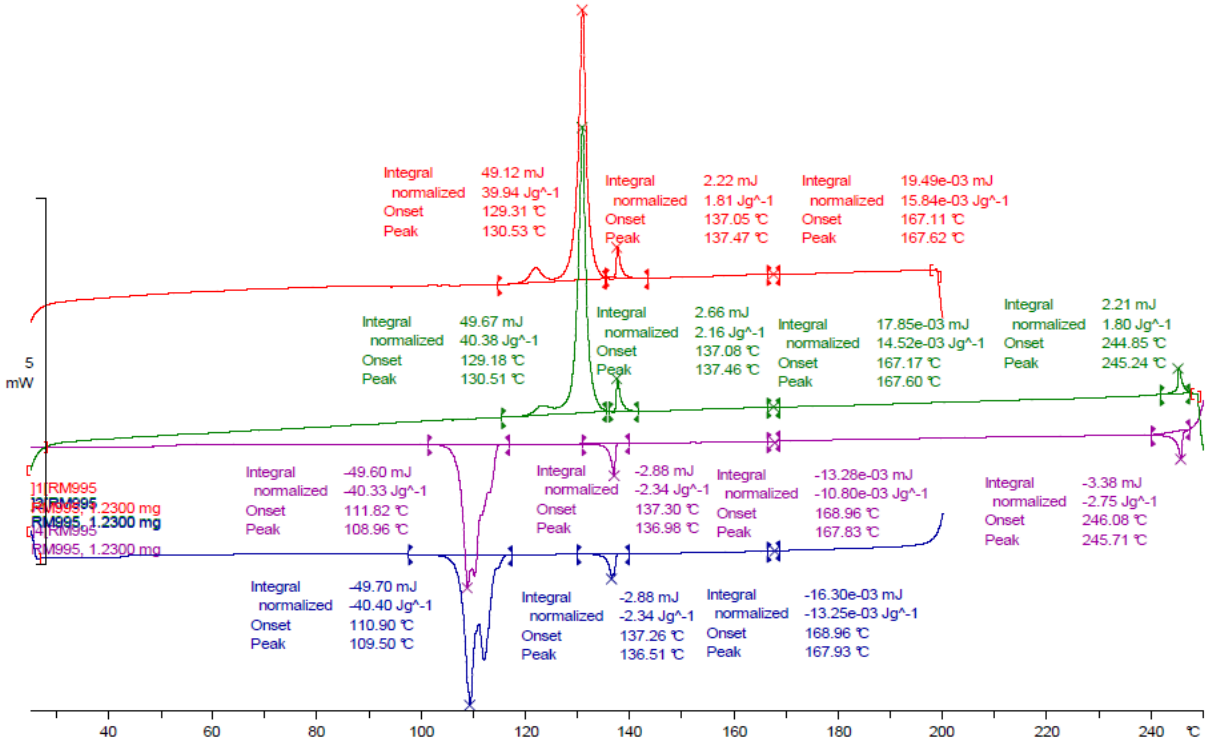

Figure SI21: DSC trace of compound **2** obtained at a heat/cool rate of 10 °C min^{-1} and a sample weight of 1.23 mg.

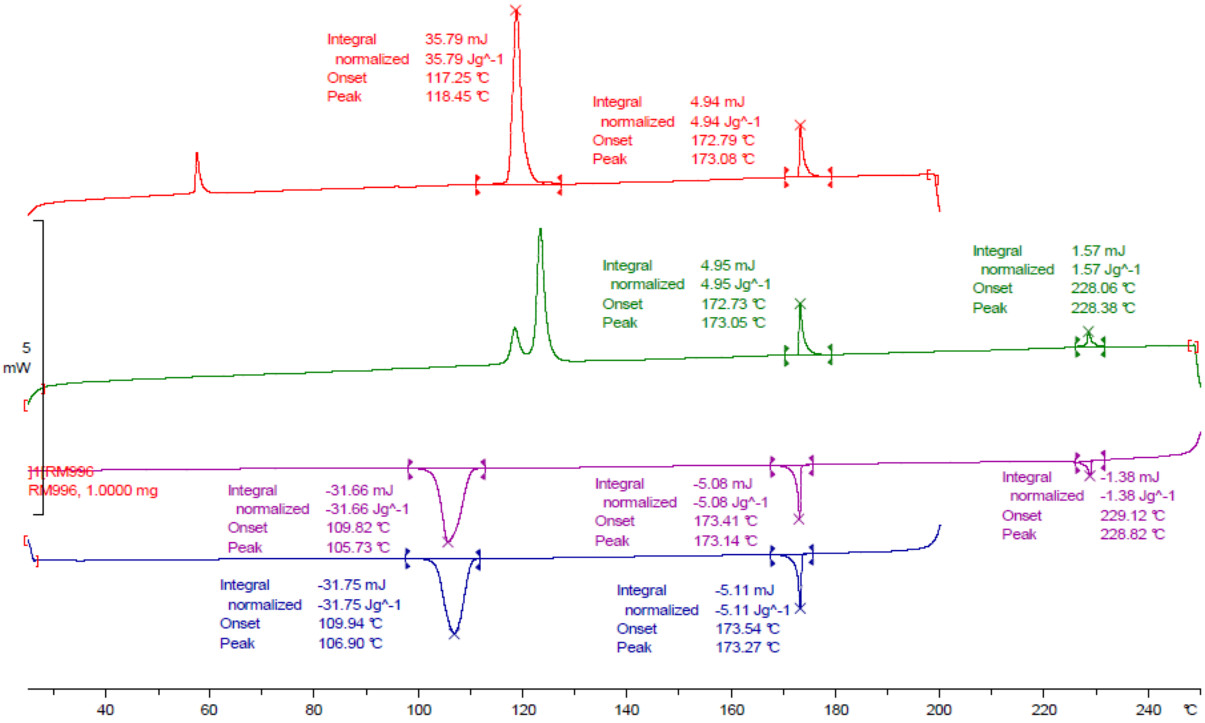

Figure SI22: DSC trace of compound **3** obtained at a heat/cool rate of 10 °C min^{-1} and a sample weight of 1.00 mg.

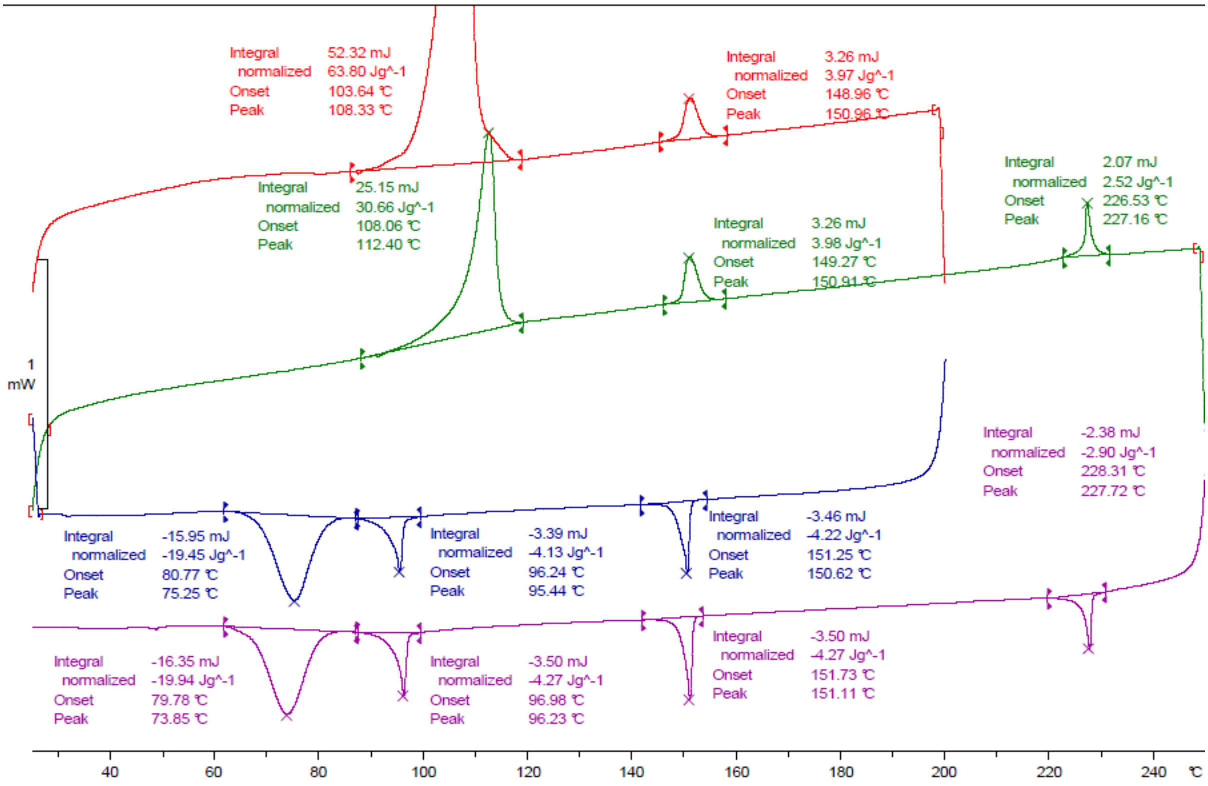

Figure SI23: DSC trace of compound **5** obtained at a heat/cool rate of 10 °C min^{-1} and a sample weight of 0.82 mg.

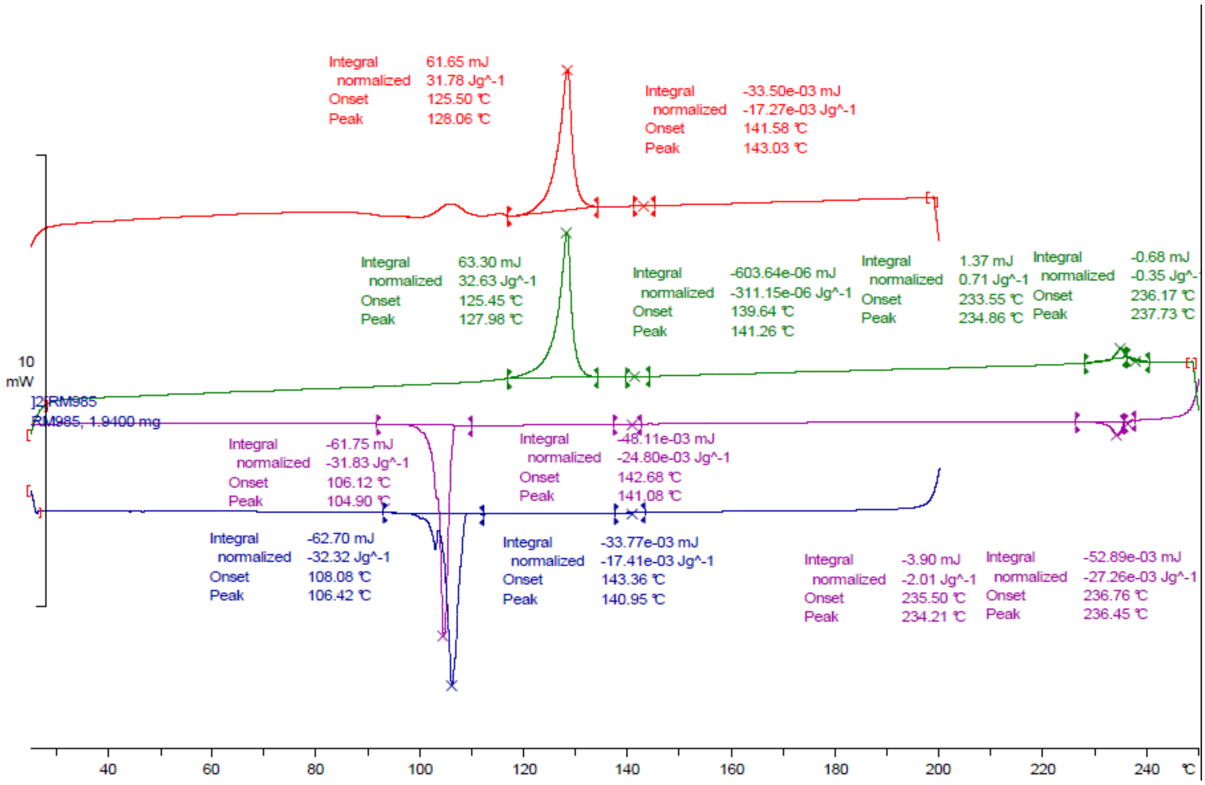

Figure SI24: DSC trace of compound **6** obtained at a heat/cool rate of 10 °C min^{-1} and a sample weight of 0.82 mg.

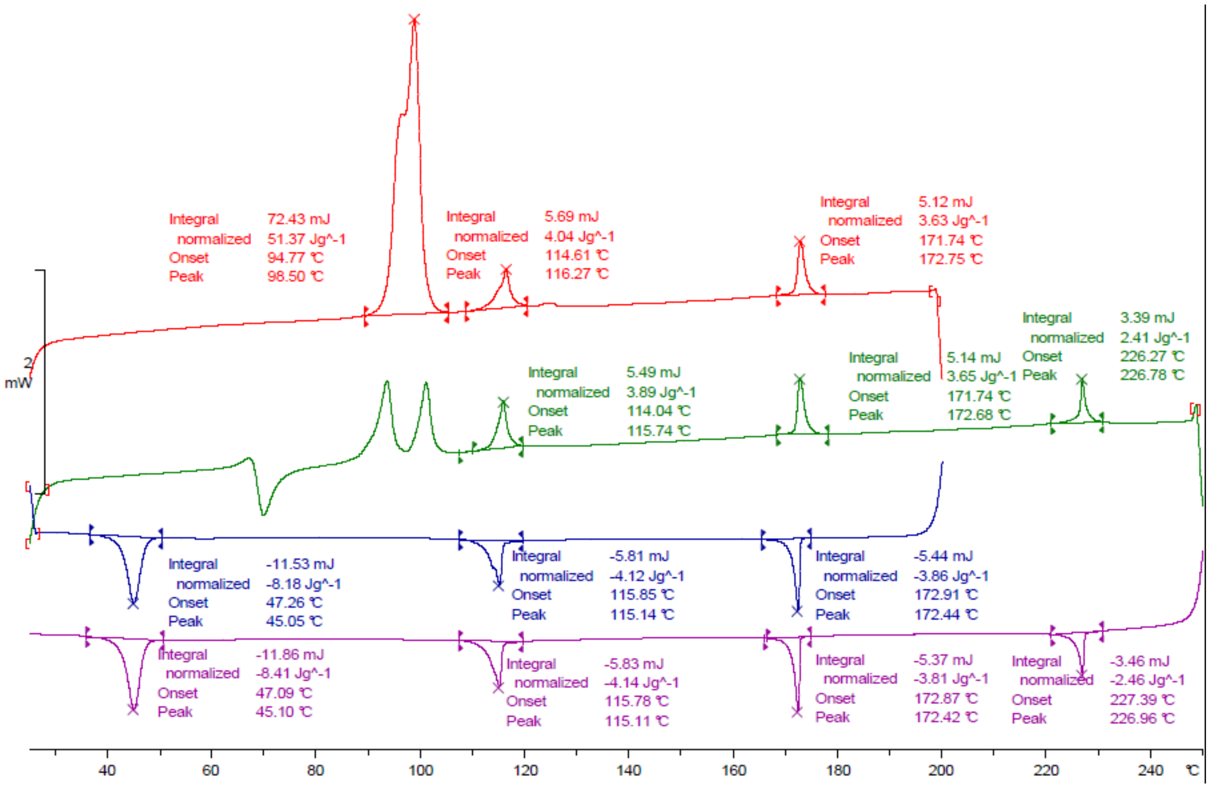

Figure SI25: DSC trace of compound **7** obtained at a heat/cool rate of 10 °C min^{-1} and a sample weight of 0.71 mg.

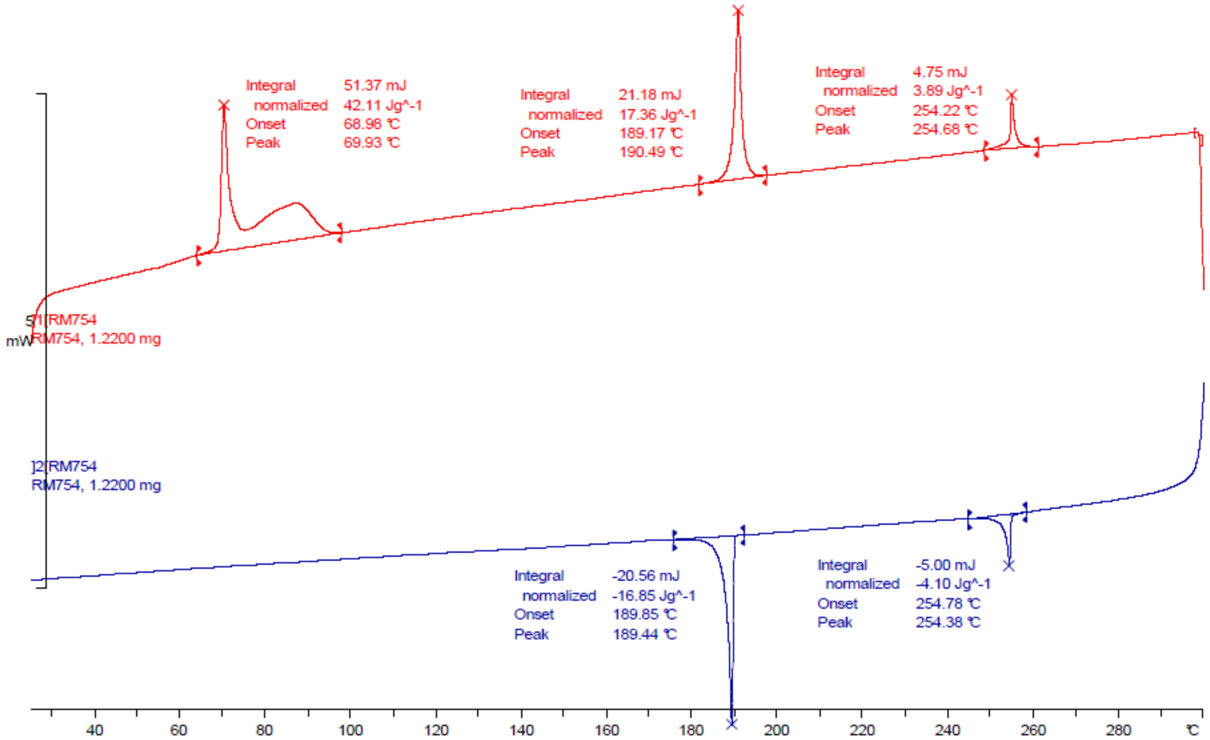

Figure SI26: DSC trace of compound **9** obtained at a heat/cool rate of 10 °C min^{-1} and a sample weight of 1.22 mg.

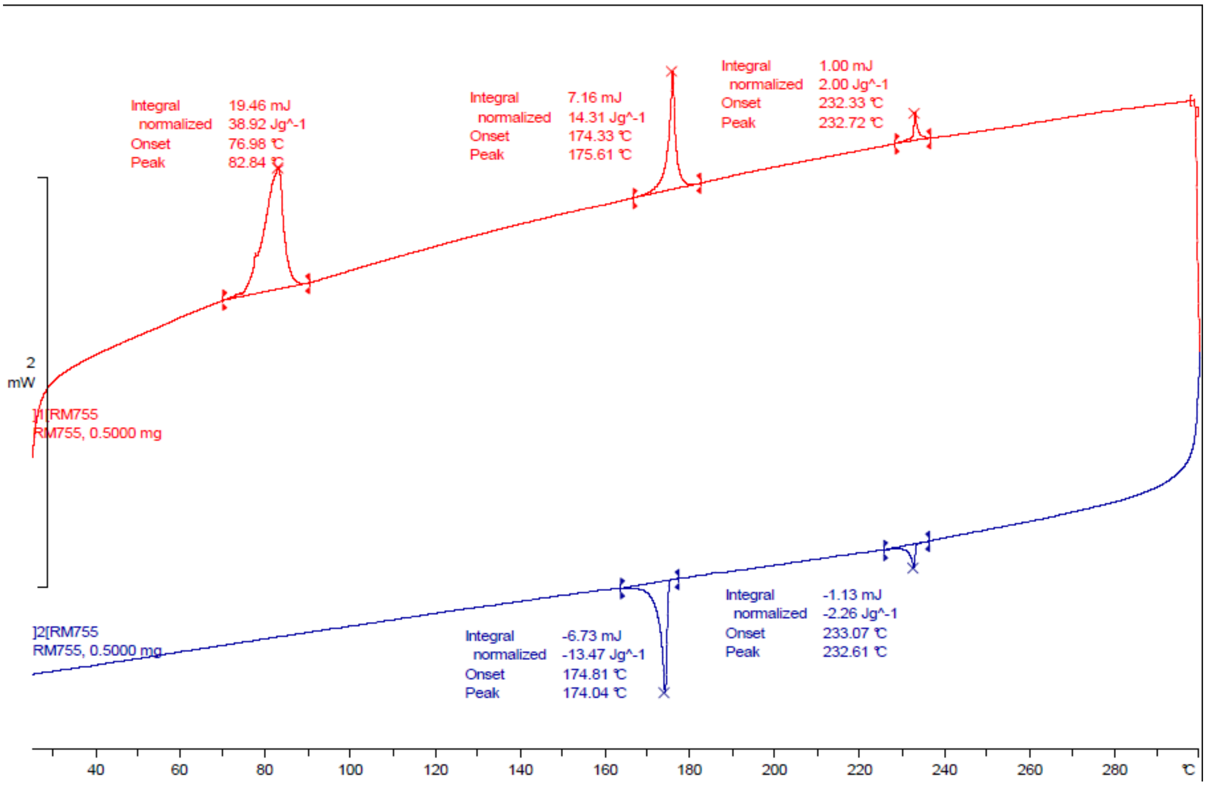

Figure SI27: DSC trace of compound **10** obtained at a heat/cool rate of 10 °C min^{-1} and a sample weight of 0.50 mg.

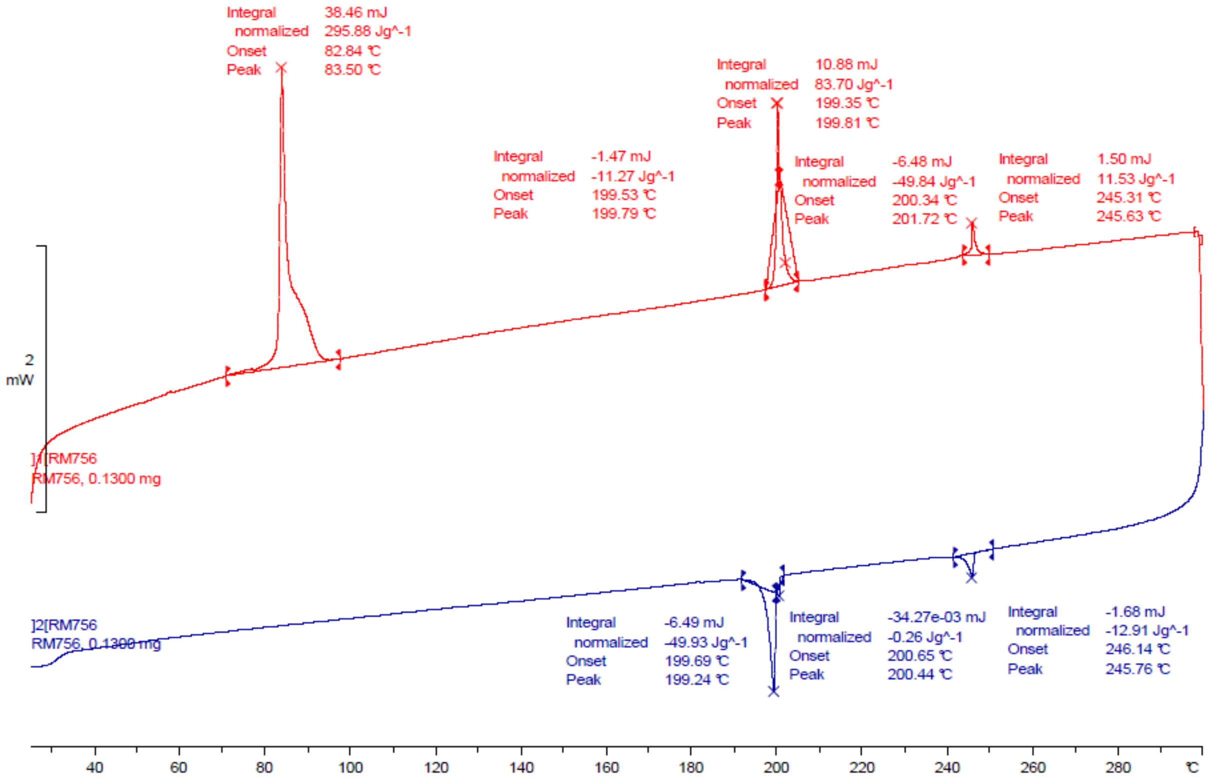

Figure SI27: DSC trace of compound **11** obtained at a heat/cool rate of 10 °C min^{-1} and a sample weight of 0.12 mg.

1.6. Tabulated Small Angle X-Ray Scattering Data

T(°C)	d-spacing (Å)
178.7	23.422
177.6	23.355
176.5	23.424
175.4	23.421
174.2	23.445
173.1	23.536
172.0	23.553
170.9	23.538
169.8	23.525
168.6	23.514
167.5	23.499
166.4	23.489
165.3	23.472
164.2	23.464
163.1	23.456
161.9	23.319
160.8	23.310
159.7	23.299
158.6	23.289
157.5	23.278
156.4	23.272
155.2	23.265
154.1	23.261
153.0	23.256
151.9	23.249
150.8	23.242
149.7	23.238
148.5	23.231
147.4	23.224

Table SI-1: Tabulated SAXS data for compound **3**

T(°C)	d-spacing (Å)	T(°C)	d-spacing (Å)
194.2	24.485	136.5	24.182
192.0	24.522	134.3	24.174
189.8	24.600	132.1	24.168
187.6	24.565	129.8	24.164
185.3	24.543	129.8	24.164
183.1	24.524	127.6	24.160
180.9	24.509	125.4	24.155
178.7	24.496	123.2	24.151
176.5	24.483	121.0	24.147
174.2	24.476	118.8	24.147
172.0	24.464	116.5	24.143
169.8	24.447	114.3	24.145
167.6	24.438	112.1	24.144
165.4	24.280	109.9	24.144
163.1	24.273	107.7	24.146
160.9	24.264	105.4	24.151
158.7	24.246	103.2	24.240
156.5	24.240	101.0	24.265
154.3	24.228	98.8	24.272
152.0	24.214	96.6	24.269
149.8	24.206	94.3	24.266
147.6	24.209	92.1	24.267
145.4	24.200	89.9	24.270
143.2	24.191	87.7	24.283
140.9	24.188	85.5	24.441
138.7	24.184	83.2	24.450

Table SI-2: Tabulated SAXS data for compound **4**

T_°C	d-spacing (Å)
187.2	23.256
184.86	23.124
182.52	23.164
180.18	23.237
177.84	23.160
175.5	23.143
173.16	23.149
170.82	23.208
168.48	23.229
166.14	23.246
163.8	23.268
161.46	23.251
159.12	23.258
118.17	23.307
105.3	23.224
93.6	23.106

Table SI-3: Tabulated SAXS data for compound **5**

T(°C)	d-spacing (Å)
169.0	22.653
167.6	22.659
166.2	22.666
164.8	22.663
163.5	22.672
162.1	22.682
160.7	22.688
159.3	22.693
157.9	22.693
156.5	22.703
155.1	22.708
153.8	22.705
152.4	22.632
151.0	21.567
149.6	21.543
148.2	21.579
146.8	21.555
145.5	21.597
144.1	21.592
142.7	21.629
141.3	21.578
139.9	21.671
138.5	21.61
137.1	21.655
135.8	21.859
134.4	21.688
133.0	21.719
131.6	21.891
130.2	21.941
128.8	21.961
127.4	22.051
126.1	22.096
124.7	22.252

Table SI-4: Tabulated SAXS data for compound **6**

T(°C)	d-spacing (Å)
193.1	22.979
190.7	22.956
188.4	23.002
186.0	23.009
183.7	23.025
181.4	23.002
179.0	23.014
176.7	23.126
174.3	23.029
172.0	23.048
169.7	23.116
167.3	23.172
165.0	23.137
162.6	23.158
160.3	23.178
158.0	23.181
155.6	23.175
153.3	23.196
150.9	23.194
148.6	23.279
146.3	23.274
143.9	23.278
141.6	23.271
139.2	23.258
136.9	23.233
134.6	23.223
132.2	23.219
129.9	23.216
127.5	23.210
125.2	23.204
122.9	23.194
120.5	23.188
118.2	23.181
115.8	23.178
113.5	23.176
111.2	23.173
108.8	23.170
106.5	23.168
104.1	23.164
101.8	23.161
99.5	23.161

Table SI-5: Tabulated SAXS data for compound **7**

T(°C)	d-spacing (Å)	T(°C)	d-spacing (Å)
227.1	23.175	179.1	23.440
224.5	23.228	177.8	23.437
221.9	23.227	176.5	23.445
219.3	23.321	175.2	23.450
216.7	23.229	173.9	23.485
214.1	23.278	172.6	23.472
211.5	23.257	171.3	23.461
208.9	23.347	170.0	23.484
206.3	23.308	168.7	23.541
203.7	23.345	167.4	23.543
201.1	23.337	166.1	23.556
198.5	23.322	164.8	23.567
196.0	23.326	163.5	23.580
194.7	23.309	162.2	23.584
193.4	23.335	160.9	23.577
192.1	23.459	159.6	23.596
190.8	23.446	158.3	23.576
189.5	23.345	157.0	23.578
188.2	23.330	155.7	23.576
186.9	23.425	154.4	23.571
185.6	23.352	153.1	23.572
184.3	23.351	151.8	23.565
183.0	23.352	150.5	23.561
181.7	23.429	149.2	23.554
180.4	23.421	147.9	23.547

Table SI-6: Tabulated SAXS data for compound **8**

t_(C)	d-spacing(Å)
215.3	24.304
214.1	24.356
212.9	24.180
211.8	24.234
210.6	24.296
209.4	23.749
208.3	24.196
207.1	24.309
205.9	24.348
204.8	24.515
203.6	24.377
202.4	23.052
201.2	23.228
200.1	23.177
198.9	23.407
197.7	23.420
196.6	23.505
195.4	23.481
194.2	23.517
193.1	23.584
191.9	23.659
190.7	23.540
189.5	23.488
188.4	23.479

Table SI-7: Tabulated SAXS data for compound **11**

T_(°C)	d-spacing (Å)
222.3	23.408
221.13	23.361
219.96	24.677
218.79	23.380
217.62	24.788
216.45	23.856
215.28	24.110
214.11	24.161
212.94	23.987
211.77	24.041
210.6	24.102
209.43	23.560
208.26	24.003
207.09	24.115
205.92	24.154
204.75	24.319
203.58	24.183
202.41	24.233
201.24	24.408
200.07	24.230
198.9	24.432
197.73	24.558
196.56	24.715
195.39	24.685
194.22	24.712
193.05	24.655
191.88	24.696
190.71	24.684
189.54	24.697
188.37	24.683

Table SI-8: Tabulated SAXS data for compound **12**

1.7. Tabulated DSC data for compounds 1, 2, 5 and 6

		Associated enthalpy (DSC cycle number)						mean	SD
No.	Transition	1	2	3	4	5	6		
1	N_{TB} - N	0.0139	0.0097	0.0091	0.0090	0.0123	0.0136	0.01127	0.0019
	N - Iso	1.3082	1.3082	1.3082	1.2973	1.2918	1.2808	1.29909	0.0096
2	N_{TB} - N	0.0107	0.0100	0.0099	0.0098	0.0108	0.0119	0.01053	0.0007
	N - Iso	1.3776	1.3818	1.3692	1.3860	1.3776	1.3860	1.37970	0.0054
5	N_{TB} - N	0.0149	0.0158	0.0144	0.0131	0.0145	0.0134	0.01436	0.0008
	N - Iso	1.3724	1.3656	1.3656	1.3589	1.3589	1.3656	1.36452	0.0043
6	N_{TB} - N	0.0126	0.0143	0.0132	0.0133	0.0139	0.0105	0.01297	0.0011
	N - Iso	1.5579	1.5525	1.5579	1.5579	1.5415	1.5360	1.55063	0.0081

Table SI-9: Tabulated associated enthalpies of transition (kJ mol^{-1}) for each DSC heat/cool cycle for compounds **1**, **2**, **5**, and **6** along with mean values and standard deviations.

References

[1] R. J. Mandle *et. al., Liq. Cryst.,* **2015**, *42*, 688-703.

[2] R. J. Mandle *et. al., Chem. Eur. J.*, **2015**, *21*, 8158-8167

[3] Gaussian 09, Revision D.01, M. J. Frisch, G. W. Trucks, H. B. Schlegel, G. E. Scuseria, M. A. Robb, J. R. Cheeseman, G. Scalmani, V. Barone, B. Mennucci, G. A. Petersson, H. Nakatsuji, M. Caricato, X. Li, H. P. Hratchian, A. F. Izmaylov, J. Bloino, G. Zheng, J. L. Sonnenberg, M. Hada, M. Ehara, K. Toyota, R. Fukuda, J. Hasegawa, M. Ishida, T. Nakajima, Y. Honda, O. Kitao, H. Nakai, T. Vreven, J. A. Montgomery, Jr., J. E. Peralta, F. Ogliaro, M. Bearpark, J. J. Heyd, E. Brothers, K. N. Kudin, V. N. Staroverov, R. Kobayashi, J. Normand, K. Raghavachari, A. Rendell, J. C. Burant, S. S. Iyengar, J. Tomasi, M. Cossi, N. Rega, J. M. Millam, M. Klene, J. E. Knox, J. B. Cross, V. Bakken, C. Adamo, J. Jaramillo, R. Gomperts, R. E. Stratmann, O. Yazyev, A. J. Austin, R. Cammi, C. Pomelli, J. W. Ochterski, R. L. Martin, K. Morokuma, V. G. Zakrzewski, G. A. Voth, P. Salvador, J. J. Dannenberg, S. Dapprich, A. D. Daniels, Ö. Farkas, J. B. Foresman, J. V. Ortiz, J. Cioslowski, and D. J. Fox, Gaussian, Inc., Wallingford CT, **2009**.

[4] L. M. Harwood, *Aldrichimica Acta,* **1985**, *18*, 25.

[5] D. S. Pedersen and C. Rosenbohm, *Synthesis*, **2001**, *16*, 2431-2434.
